# Synthesis, properties, and application of the new nanocatalyst of double layer hydroxides in the one-pot multicomponent synthesis of 2-amino-3-cyanopyridine derivatives

**DOI:** 10.1038/s41598-023-27940-6

**Published:** 2023-01-28

**Authors:** Sarieh Momeni, Ramin Ghorbani-Vaghei

**Affiliations:** Department of Organic Chemistry, Faculty of Chemistry, Bu-Ali Sina University, Hamedan, Iran

**Keywords:** Chemistry, Materials science

## Abstract

A new heterogeneous nanocatalyst LDH@3-chloropyltrimethoxysilane@1,3-benzenedisulfonyl amine@Cu (LDH@TRMS@BDSA@Cu) was synthesized and confirmed by analyzes such as Fourier transform infrared spectroscopy, Field Emission Scanning Electron Microscopy, energy scattered X-ray spectroscopy (EDX), elemental mapping, X-ray diffraction analysis, heat gravity/heat derivatization (TGA) and differential scanning calorimetry. The newly synthesized nanocatalyst effectively catalyzed the reaction between different aryl aldehydes, malononitrile, different acetophenones and ammonium acetate in solvent-free conditions and they were converted into 2-amino-3-cyanopyridine derivatives with high efficiency. The reaction showed advantages such as simplicity, high stability, environmental friendliness, excellent efficiency and short time. Also, this catalyst is recyclable and was recycled 4 times without losing significant catalytic power.

## Introduction

In recent years, two-dimensional nanomaterials have been widely studied and used as attractive candidates for the construction of heterogeneous solid catalysts, electrodes, adsorbents, metal-sulfur batteries, etc^1–3^. Double-layered hydroxides known for more than a decade, are abundant in nature and easily extracted, and represent a large class of anion- and cation-exchangeable layered structures with the general formula [M^2+^ (1-x) Mx^3+^(OH)^2^ is](An^-^) x/n.zH_2_O]. Metal cations that are used in divalent and trivalent form are Mn^2+^, Fe^2+^, Mg^2+^, Co^2+^, Zn^2+^, Ca^2+^ and Mn^3+^, Fe^3+^, Co^3+^, Cr^3+^, Al^3+^ and the anions used often contain carbonate, bromide, chloride or are nitrates^4–6^. There are various methods for the synthesis of LDHs, among which can be mentioned ion exchange, hydrothermal and co-precipitation methods. LDHs are neutral materials, the middle parts of the anion and the layers themselves have a positive charge, which have many applications in different fields due to their easy synthesis and the ability to replace and modify the hydroxide layers, which have attracted a lot of attention from researchers, such as adsorbents^7^, catalyst bases^8, 9^, anion exchangers, water electrolysis^10^, energy storage^11, 12^, sensors. The easy separation of heterogeneous catalysts such as bilayer hydroxides provides an easy and fast route for catalyst recovery, and catalyst recovery is valid both green chemistry and economically. Due to the unique characteristics and interesting physical properties of copper iodide, including high optical transparency with wide band gap, high conductivity with unusual diamagnetic behavior, wide band slit, synthesis at low temperature has been investigated in many research works^13–15^. Copper iodide crystallizes with three different phases α, β and γ with temperature changes during synthesis, which at a temperature higher than 407 °C is the cubic alpha phase, which at a temperature above 369 °C is the hexagonal beta phase, and at low temperatures, copper iodide with high crystallinity is the cubic gamma phase, which is a type of semiconductor in which iodide ions tetrahedrally surround copper ions. The applications of this nanocopper can be mentioned such as diodes, solar cells, semiconductor patterns, and organic catalysts^16^.

A useful strategy for the synthesis of heterocyclic compounds such as pyridines is multicomponent reactions involving at least three components to produce the product with all the starting materials involved, which is cost-effective in terms of green chemistry^17, 18^. Heterocyclic compounds such as pyridine due to their unique biological and medicinal properties such as antibacterial, anticonvulsant, anti-malarial, antioxidant, anti-diabetic agent, anti-inflammatory, analgesic, anti-cancer, anti-tumor, liver protector, anti-atherosclerotic, anti-fungal and anti-pest properties have attracted the most attention among heterocyclic compounds. Compounds containing the 2-amino-3-cyanopyridine framework are used as useful therapeutic precursors in the medical field due to their biological activity^19–21^. Various synthesis methods have been reported for their synthesis, in which the multicomponent reactions of ammonium acetate, malononitrile, acetophenone, and aldehydes are the most important synthesis routes. A wide range of multicomponent syntheses have been reported by different catalysts, including: Boric acid sulfate nanocatalysts^21^, HBF_4_^22^, microwave facile^23^, Amberlyst-15^24^, salicylic acid^4^, MNPs CoFe_2_O_4_@SiO_2_-SO_3_H^25^, nano solid magnetic acid, Fe_3_O_4_^26^, Fe_3_O_4_@g-C_3_N_4_-SO_3_H^27^, Fe_3_O_4_@SiO2@(CH_2_)_3_NH^28^, (CH_2_)_2_O_2_P(OH)_2_^29^, poly N,N-dimethylaniline-formaldehyde^30^, Copper Nanoparticles on Charcoal^31^, Fe_3_O_4_@Niacin^32^, Bu_4_N^+^Br^-18^, Cu@imineZCMNPs^17^. However, simpler and milder methods for their synthesis are still valuable. However, simpler and milder methods for their synthesis are still valuable. According to the points mentioned, the aim of the study is to develop fast and simple methods based on green chemistry, recovery and reuse of catalyst for the synthesis of new derivatives of cyanopyridines. Here, we succeeded to make a unique catalyst with 1,3-benzenedisulfonyl amide (BDSA) ligand placed on LDH to immobilize copper iodide nanoparticles (LDH@TRMS@BDSA@Cu) as a new and efficient nanocatalyst. For the one-pot synthesis of four-component 2-amino-3-cyanopyridine, the reaction was used between different aryl aldehydes 1, malononitrile 2, different acetophenones 3 and ammonium acetate 4 in mild conditions without solvent (Supplementary file 1).

## Experimental

### General

All chemical materials in this work were purchased from Merck company, and used without further purifications. Fourier transform infrared (FT-IR) spectroscopy was obtained on a Perkin Elmer GX FT-IR spectrometer in the range of 4000–400 cm^−1^. ^1^H NMR and ^13^C NMR spectra were recorded in DMSO-*d*6 solvent on Bruker BioSpin GmbH 300 MHz FT NMR spectrometers. Melting points of the samples were identified in open tubes on a BUCHI 510 apparatus. The structure of the new LDH@TRMS@BDSA@Cu catalyst was identified using FTIR, FESEM, XRD, EDX, MAPPING, TGA, and DSC analysis. Field Emission Scanning Electron Microscopy (FESEM) was performed with FE-SEM TESCAN MIRA3 instrument and X-ray Diffraction (XRD) patterns of samples were recorded with a philips PW1730 in a range from 10 and 90° (2θ). Energy dispersive X-ray (EDX) analysis of the synthesised catalyst obtained by the EDAX-EDS apparatus. Thermo-gravimetric analysis (TGA) was recorded on a TGA-DTA apparatus in the N_2_ at a heating rate of 10 °C min^−1^ in the temperature range of 25–600 °C, differential scanning calorimetry (DSC) was record on a DSC apparatus. The reaction progress and the purity of the products were assessed by thin-layer chromatography (TLC) with silica gel plates.

### General procedure for preparation of LDH

Zn-Cr -LDH was synthesized according to the previous command, which is briefly described below. Salts of Cr (NO_3_) _3_.9H_2_O and Zn (NO_3_) _2_·6H_2_O with a molar ratio of 2/1 were dissolved in deionized water; during intense stirring pH of the solution was reached up 11.5 with 2 M NaOH aqueous solution, then the resulting solution was placed at the same temperature for 18 h. The obtained green compound was filtered, rinsed with distilled water, and dried in an oven at 60 °C for 24 h.

### General procedure for synthesis of LDHs coated by 3-chloropropyltrimethoxysilane (LDHs@TRMS)

To activate LDH from 3-chloropropyltrimethoxysilane was used. For this purpose, 1 g of the LDH synthesized in the previous step was dissolved in 50 mL of toluene, then 2 mL of 3-chloropropyltrimethoxysilane was added, and the resulting solution was refluxed with constant stirring for 12 h. The precipitate was then collected with filter paper, washed with toluene and ethanol several times, and placed in an oven at 50 °C for drying.

### Procedure for synthesis of 1,3-benzenedisulfonyl chloride

First, PCl_5_ (16.5 mmol), as chlorination agent, was added to a container containing of 1,3-benzenedisulfonic acid disodium salt (5.00 g, 18 mmol), then sterilized and heated to 65 °C, and the reaction was continued for 2 h; after completion of the reaction, dry ice (100 g) and chloroform (100 ml) were added to the reaction vessel, and the organic layer was separated^33^.

### Procedure for synthesis of ligand: 1,3-benzenedisulfonyl amide (BDSA)

In a 50 mL flask containing 1 g of 1,3-benzenedisulfonyl chloride and 5 mL of amide were added and placed under reflux conditions for 12 h. After the reaction was completed, the lid of the container was closed with paraffin and placed 0 °C to synthesize the desired crystals, and then the desired crystals were collected and dried.

### Procedure for synthesis of LDH@TRMS@BDSA

The activated double-layer hydroxide with 3-chlorotrimethoxysilane, was dispersed homogeneously in 50 mL of toluene for 15 min by ultrasonic device, then was refluxed for 24 h. The resulting mixture was collected with filter paper and washed with distilled water and ethanol several times, and dried in an oven at 60 °C for 24 h.

### Procedure for synthesis of LDH@TRMS@BDSA@Cu

According to the description of the article^34^, nano copper was synthesized. For loading Cu NPs, to a container of content LDH in the ethanol (0.5 g in 20 mL) which was dispersed for 5 min, 0.3 g of Cu NPs was added into the mixture and was refluxed for 12 h. Finally, the LDH nanoparticles were collected through centrifugation, washed with distilled water and dried under vacuum conditions at 60 °C for 24 h.

### General procedure for synthesis of one-pot four-component 2-amino-3-cyanopyridine derivatives

To a vial containing a mixture of acetophenone (1.0 mmol), malononitrile (1.0 mmol), ammonium acetate (2.5 mmol) as a source of nitrogen and aromatic aldehydes (1 mmol) and LDH@TRMS@BDSA@Cu nanocatalyst (0.05 g) entered. The mixture was stirred at 60 °C in an oil bath and the reaction was followed by TLC. After formating the desired composition, the mixture was cooled to room temperature, then hot ethanol (2 mL) was added to the reaction vessel. The LDH@TRMS@BDSA@Cu nanocatalyst was easily separated from the solution by centrifugation. After evaporating the solvent, a pure composition was obtained in ethanol.

## Results and discussion

### Properties of catalyst

**Figure 1:**
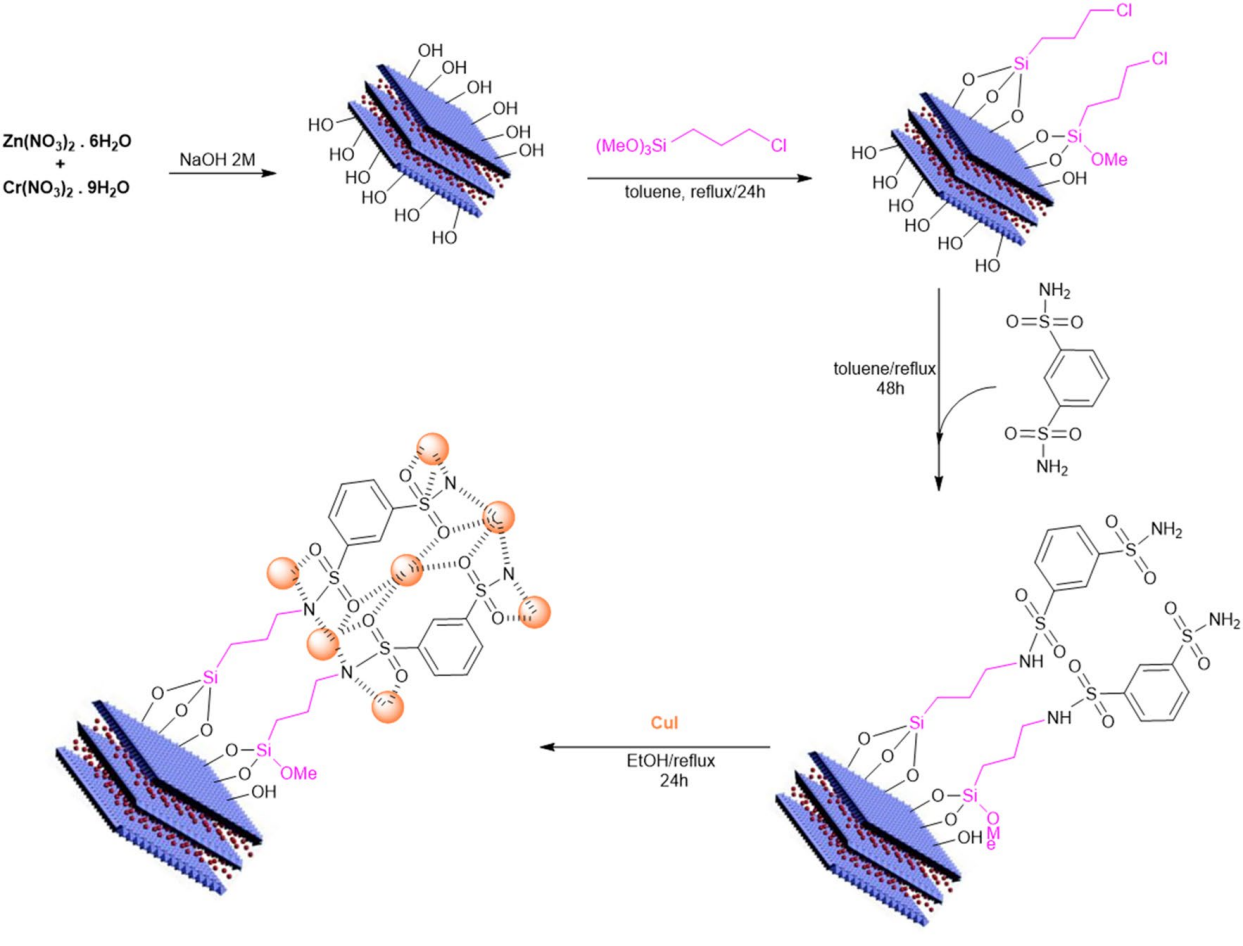
Process of synthesis of new heterogeneous nanocatalyst (LDH@TRMS@BDSA@nCu).

**Figure 2:**
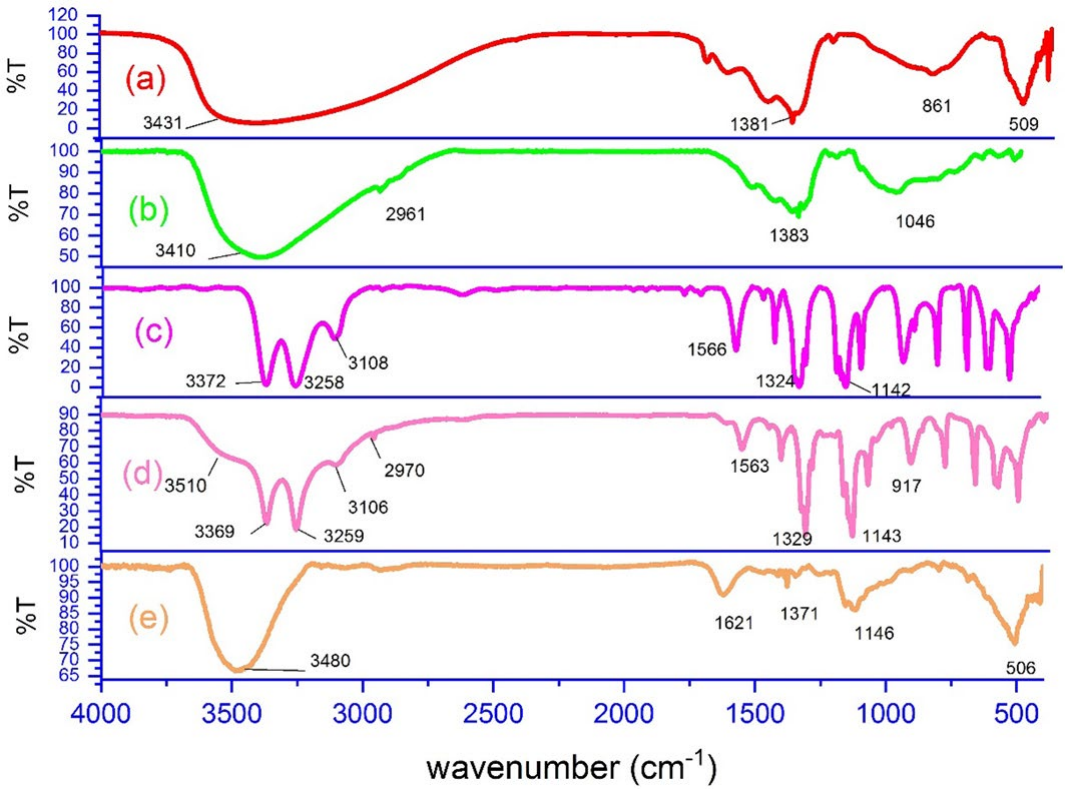
FTIR of (**a**) LDH, (**b**) LDH@TRMS, (**c**) DSA, (**d**) LDH@TRMS@DSA and (**e**) LDH@TRMS@DSA@nCu.

The synthesis steps of the new LDH@TRMS@BDSA@Cu nanocatalyst are shown in Fig. 1. As can be seen, copper nanoparticles were stabilized on the LDH surface using 1 and 3 benzene disulfonylamide, and the synthesized nanocatalyst was characterized by Fourier transform infrared (FT‐IR), Field Emission Scanning Electron Microscopy (FESEM), energy‐dispersive X‐ray spectroscopy (EDS), X‐ray mapping, X‐ray diffraction (XRD), differential scanning calorimetry (DSC) and thermal gravimetric analysis (TGA). FT-IR spectroscopy was first studied to confirm the structure of the LDH@TRMS@DSA@Cu nanocatalyst. Figure 2 shows the FT-IR spectra of (a) LDH, (b) LDH@TRMS, (c) DSA, (d) LDH@TRMS@BDSA and (e) LDH@TRMS@BDSA@Cu.

An extensive absorption peak in the region of approximately 3431 cm^-1^ indicates tension vibration of O–H on LDH surfaces. Its bending vibration was observed at about 1600 cm^-1^. Absorption peak 1381 cm^−1^ is a characteristic of an uncoordinated nitrate anion that belongs to other layered hydroxides containing interlayer nitrate groups. Absorption peaks less than 1020–500 cm^−1^ are due to network vibrations of LDHs (M–O, O–M–O). Bands between 850 and 1017 cm^−1^ can be attributed to M–O tensile modes, and a band of about 509 cm-^1^ can be attributed to O-M–O tensile modes (part (a) of Fig. 2). The absorption peak observed at 2961 cm^−1^ corresponds to the tensile vibrations of the C–H group of the alky of 3-chlorotrimethoxysilane group (part (b) of Fig. 2). The tensile and bending vibrations of NH are observed at peaks of 3372, 3258 and 1566 cm^−1^. Also, the peaks in areas 1324 and1142 cm^-1^ belong tension vibration of group S=O. In Part )d) of Fig. 2, it is shown that the peak in region 3510 cm^−1^ belongs to the tensile vibration of O–H on the surface of LDH, the peak in region 2970 cm^−1^ corresponds to the tensile vibration of C–H, and the tensile vibrations of sulfonyl in region 1329 and 1143 cm^−1^ confirms BDSA coordinates to LDH@TRMS. In addition, according to part (e) of Fig. 2 that the peaks related to NH have rounded. Also, the intensity of the peaks related to S=O have decreased, indicating their overlap with nanomaterials.

**Figure 3:**
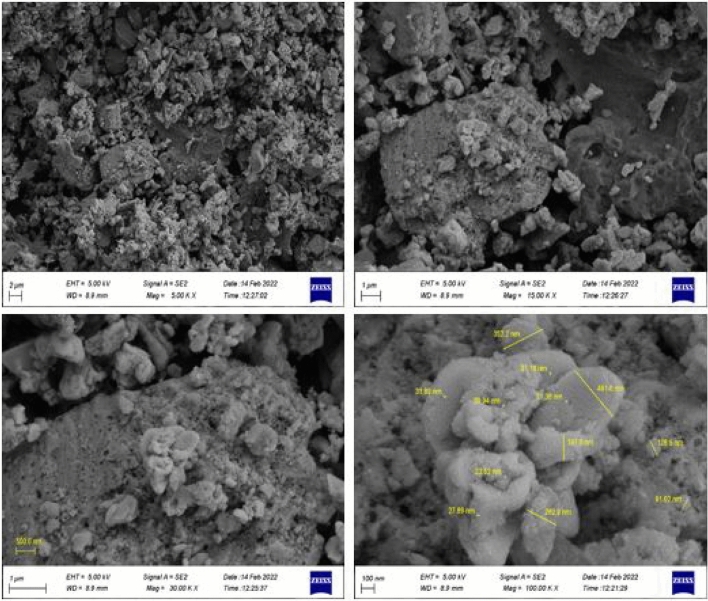
FESEM images of LDH@TRMS@BDSA@nCu.

**Figure 4:**
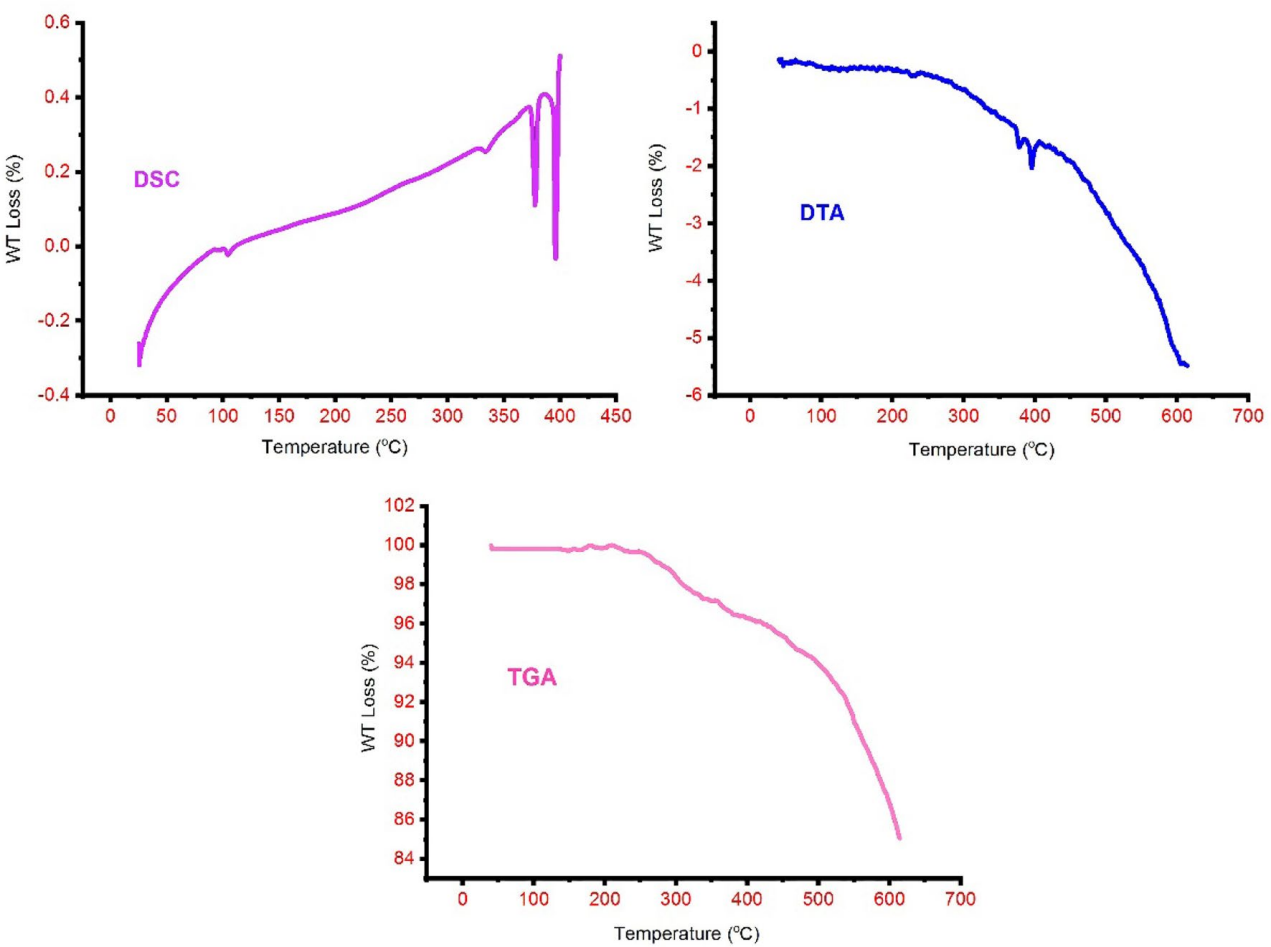
DSC, DTA and TGA analysis curves of LDH@TRMS@BDSA@Cu.

To obtain more information about the synthesized nanoparticles, the morphology and size of LDH and MNPs of LDH@TRMS@BDSA@nCu were examined by the FESEM method. Figure 3 shows that the LDH particles are in the form of sheets that are stacked on top of each other, indicating that the catalyst has grown like a plate. The prepared copper nanoparticles are shown according to image 3 to be almost spherical and fixed on LDH. TGA thermal gravimetric analysis was used to show the thermal stability of LDH@TRMS@BDSA@Cu (Fig. 4). With increasing temperature, several ranges of mass reduction were observed. The first partial decrease in mass at temperatures below 200 °C is related to water coming out of the sample, which is mainly in the layers. At higher temperatures, about 370 weight reductions have been observed, which is related to the decomposition and dissolution of organic groups. These cases confirm that the LDH@TRMS@BDSA@Cu catalyst is stable at or below 370 °C. In addition, the DSC and DTA curves showed that the LDH nanocatalyst is stable below 350 °C.

**Figure 5:**
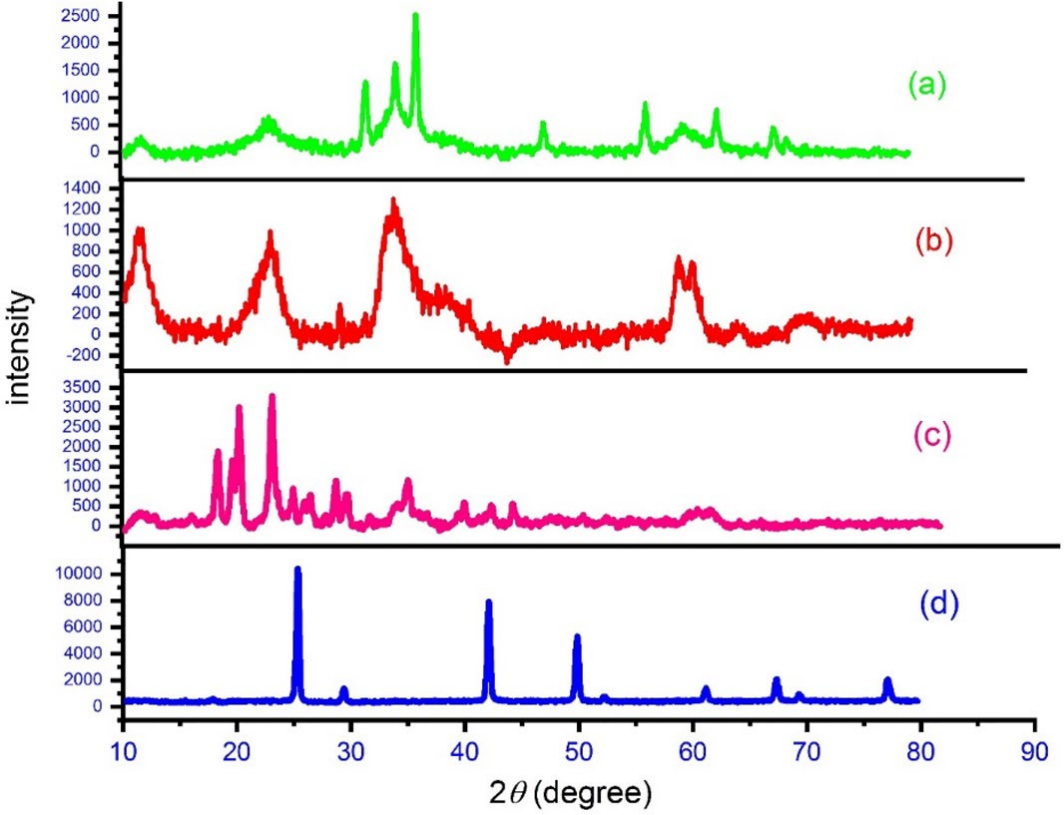
XRD analysis of (**a**) LDH, (**b**) LDH@TRMS, (**c**) LDH@TRMS@BDSA and (**d**) LDH@TRMS@BDSA@Cu.

XRD model was used to study the crystallinity and particle size of the catalyst .The XRD patterns of different stages of nanocatalyst synthesis are shown in Fig. 5. The XRD patterns of LDH, LDH@TRMS, LDH@TRMS@BDSA, and of LDH@TRMS@BDSA@Cu peaks in regions 10, 20, 25, 40, 50, 60, 70 and 80 indicate that these specimens have high crystallinity and long-range order. They also show that DSA and nano-copper are stabilized on LDH^34^.

**Figure 6:**
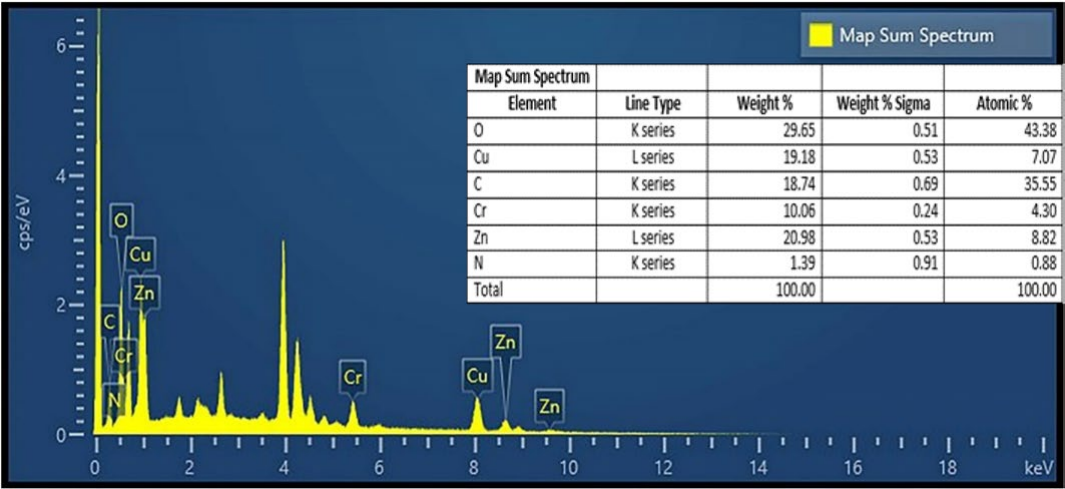
EDX analysis of the novel catalyst.

**Figure 7:**
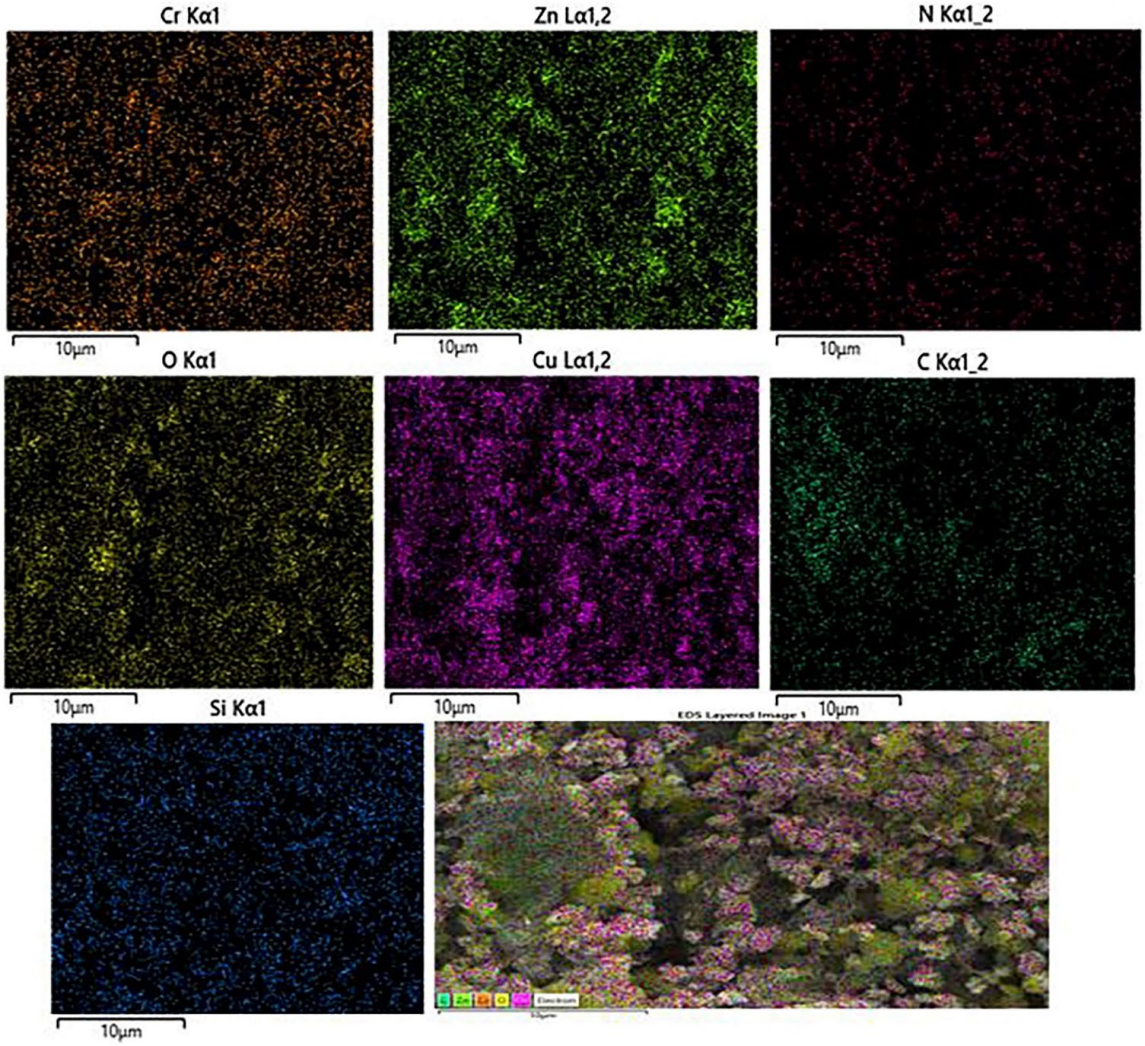
Elemental mapping (EDX) of Cr (orange); Zn (bright green); N (red); O (yellow); Cu (purple;C (dark green), and Si(blue) atoms for LDH@TRMS@BDSA@Cu.

EDX analysis indicated the chemical properties and elements present in the synthesized catalyst. The analysis results showed the successful formation of intermediates with the presence of zinc, chromium, oxygen, carbon, nitrogen, and nano-copper atoms in the catalyst structure (Fig. 6). In addition, Fig. 6 shows Weight %, Weight % Sigma, and Atomic % related to Cu, O, N, Cr, Zn, and C elements. The elemental composition of the catalyst synthesized by the elemental map confirmed the presence of the mentioned elements and showed a uniform distribution of these elements in the composition (Fig. 7). The results of these Figures confirm the presence of the mentioned elements in the catalyst structure.

### Catalytic activity

After confirming the new nanocatalyst, we synthesized 2-amino-3-cyanopyridine derivatives to evaluate its catalytic activity. In the first step, to evaluate the optimal synthesis conditions of malononitrile (1.0 mmol), ammonium acetate (2.5 mmol), 4-acetophenone (1.0 mmol), and 4-Cl-benzaldehyde (1.0 mmol) as substrates of the model were selected. As shown in Table 1, the model reaction was performed in the presence of various solvents, including water, ethanol, methanol, and acetonitrile, in solvent-free conditions at 60 °C in the presence of the catalyst. The results showed that the solvent could have a good effect on the product yield, but the best efficiency and short reaction time in solvent-free conditions in the presence of 0.05 g were obtained from LDH@TRMS@BDSA@Cu nanocatalysts. LDH@TRMS@BDSA@Cu was a suitable catalyst for synthesizing 2-amino-3-cyanopyridine with a short reaction time and high efficiency. Then, the model's reaction is investigated using different values of LDH@TRMS@BDSA@Cu under solvent-free conditions at different temperatures, and the results are given in Table 1.

**Table 1:** Optimization of reaction conditions for synthesis of 2-amino-4-(4-chlorophenyl)-6-phenylnicotinonitrile.

Entry	Solvent	Load of catalyst (mg)	Temperature (°C)	Time (min)	Yield (%)
1	–	–	100	120	40
2	–	10	80	45	70
3	–	20	70	28	80
4	–	35	70	18	85
5	–	50	70	8	95
6	–	50	80	7	96
7	–	50	60	9	92
8	EtOH	50	Reflux	80	50
9	MeOH	50	Reflux	85	50
10	CH3CN	50	Reflux	70	65
11	H2O	50	Reflux	80	20
12	EtOAc	50	Reflux	85	20

According to Table 1, in the absence of LDH@TRMS@BDSA@Cu nanocatalysts, the reaction was performed at a longer time, higher temperature, and with lower efficiency. Different temperatures were examined (from r.t to 90 °C), and the results showed that 60 °C was the highest efficiency and the shortest reaction time. After optimization, the optimal reaction conditions for the preparation of 2-amino-3-cyanopyridine derivatives were performed using various aryl aldehydes and acetophenones with donor or electron donor groups (Table 2). Based on the results summarized in Table 2, all 2-amino-3-cyanopyridine derivatives were easily synthesized with excellent yield. Which confirmed the very high catalytic activity of LDH@TRMS@BDSA@Cu nanocatalysts for the synthesis of 2-amino-3-cyanopyridine.

**Table 2:**
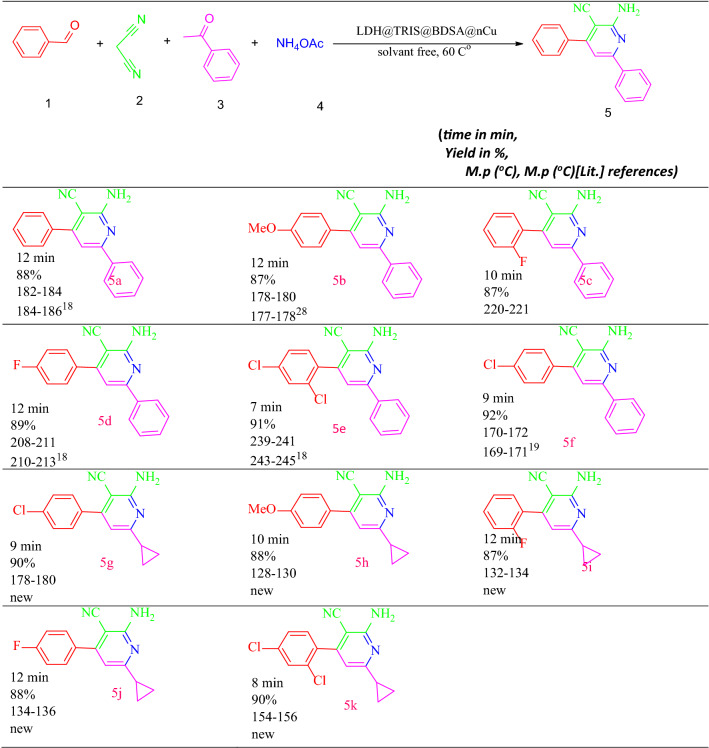
Synthesis of 2‐amino‐4,6‐diphenylnicotinonitrile derivatives under optimum conditions.

^a^Reaction conditions: arylbenzaldehyde (1 mmol), acetophenone derivatives (1 mmol), malononitrile (1 mmol, 0.066 g) and ammonium acetate (2.5 mmol, 0.231 g). ^b^Isolated yield.

**Figure 8:**
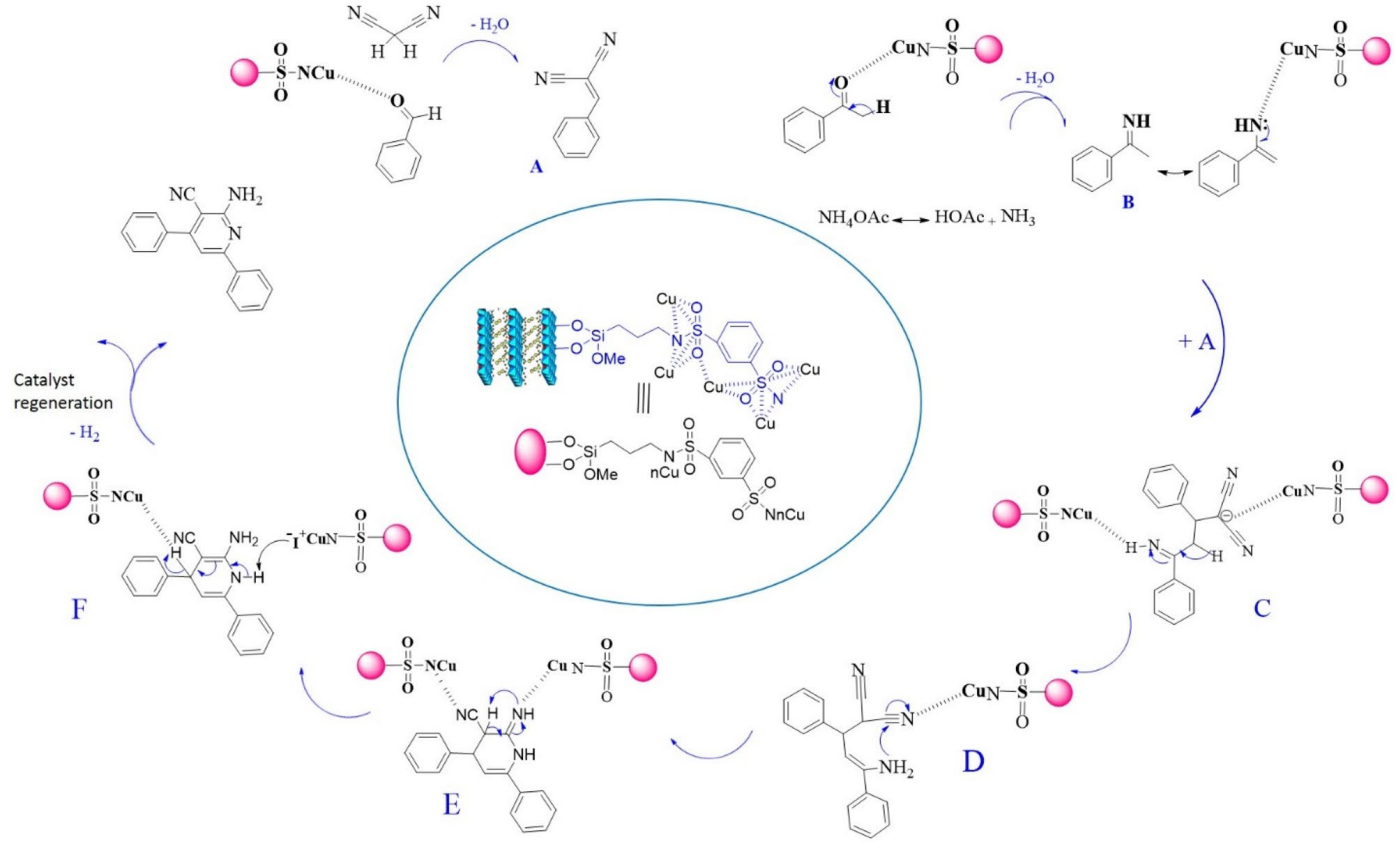
Suggested mechanistic for the synthesis of 2-amino-4,6-diphenylnicotinonitrile derivatives.

According to previous studies in the literature^28, 32^, a proposed mechanism catalyzed by LDH@TRMS@BDSA@Cu for the synthesis of 2-amino-3-cyanopyridine is shown in Fig. 8. First, the interaction of the nanomaterials on the LDH@TRMS@BDSA@Cu catalyst with the electrons of the oxygen atom in the benzaldehyde provides an active electrophilic site to attack malononitrile. The reaction between active aldehydes 1 and malononitrile 2 produces an intermediate of A arylidene malononitrile. Ammonium acetate 4, on the other hand, reacts with active acetophenones 3 to form an intermediate of enamine B. In the next step, the reaction between intermediates A and B (arylidene malononitrile A to enamine B), which takes place in the form of an increase of Michael, formed the intermediate C. Subsequent cycling/isomerization/aromatization steps were performed, which led to the formation of the desired products. And provided only one detachable product based on TLC analysis.

The structure of the reaction product was determined based on spectral data of FTIR, CNMR and HNMR as 5 g. The FT-IR spectra of the three sharp peaks in regions 3419, 3317, and 3168 cm^-1^ are related to the vibrational frequency of group NH_2_, and the peaks of region 3000 are related to the vibrational frequency of C–H aromatic and aliphatic, and the sharp peak in region 2208 is related to the functional group CN and the vibrational frequency of cyanide appeared in 1646. In the HNMR spectrum, the composition of peaks related to ring hydrogens in regions 7–8 appears. The chemical displacement in region 6.89 with integral 2 is related to amine hydrogens. The peak of region 6.73 is related to the hydrogen of the pyridine ring, and the peaks of region 1–2 are related to the aliphatic CH of the cyclopropyl ring, which appears as a multiple with an integral, and the peaks of region 2.2 are related to the aliphatic CH, a binary with integral 1. In the ^13^CNMR spectrum, the peak 171 corresponds to the carbon of the pyridine ring attached to the cyclopropyl ring, and the peak in region 160 corresponds to the carbon attached to the amine group. Aromatic carbons have appeared in the range of 146–85, and aliphatic carbons have peaked well in the range of 22–36.

**Figure 9:**
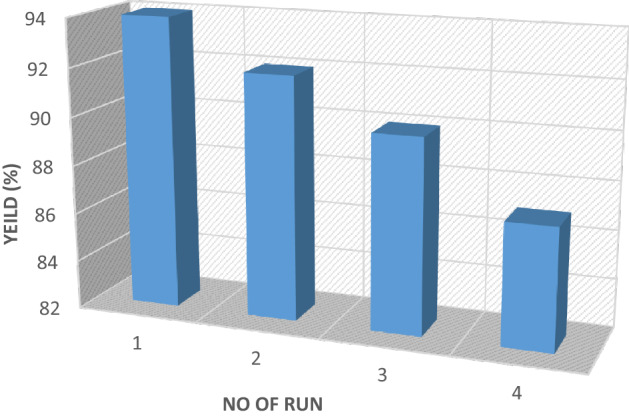
Recycling of the LDH@TRMS@BDSA@Cu nanoparticles for the reaction of the model.

Finally, LDH@TRMS@BDSA@Cu nanoparticles were isolated by simple extraction and reused for subsequent execution. This process can be repeated four times without an obvious efficiency change (Fig. 9). However, in Fig. 9 was observed a decrease in the reaction yield after four recycling and reuse of the catalyst in the synthesis reaction of 5a, which may be due to the loss of some catalyst NPs during separation, aggregation, etc.

**Figure 10:**
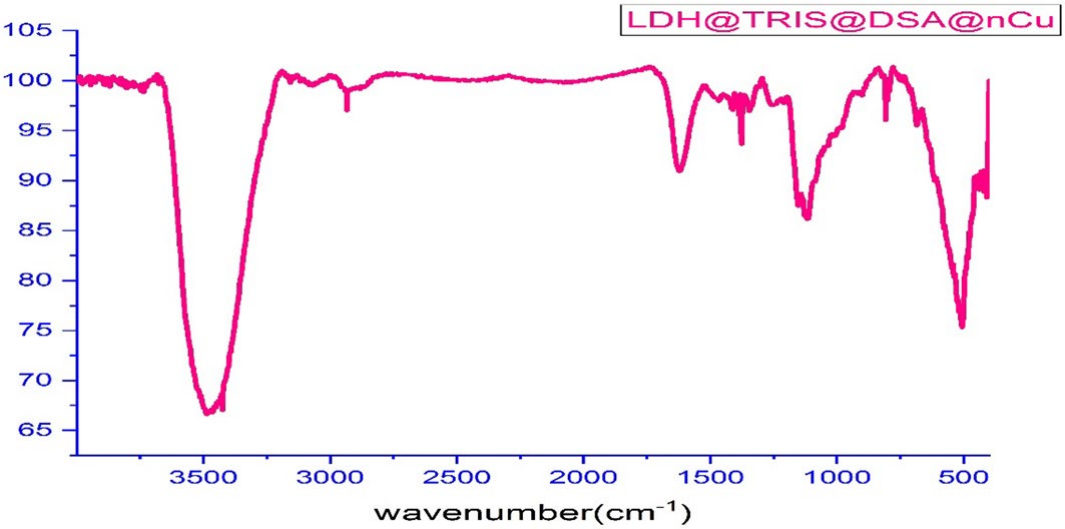
FTIR analysis of the recycled LDH@TRMS@BDSA@Cu nanocatalyst.

In the first run, 94% of LDH nanoparticles were recovered, and the purity and structure of the recovered LDH@TRMS@BDSA@Cu nanoparticles based on the FT-IR result remained unchanged (Fig. 10). Catalyst recycling and reuse of green chemistry cases were examined for the new catalyst. In this regard, recovery, and reusability of LDH@TRMS@BDSA@Cu nanoparticles under the optimal reaction of malononitrile (1.0 mmol), ammonium acetate (2 mmol), 4-acetophenone (1.0 mmol), and 4-Cl benzaldehyde (1.0 mmol) was performed in solvent-free conditions at 60 °C using 0.05 gr of catalyst. For this purpose, after each cycle, the nanocatalyst was separated from the reaction solution using centrifugation and washed several times with ethanol, dried at vacuum 60 °C, and used again in the next cycle. The catalyst can be reused according to Fig. 9 for four consecutive cycles, which show the same activity for each reaction cycle without significantly reducing its catalytic activity.

### Comparison

Based on Table 3, the catalytic protocol of LDH@TRMS@BDSA@Cu was compared with the reported protocols for the synthesis of 2-amino-3-cyanopyridines, which showed the results of this new catalyst as a new, green, and efficient nanocatalyst with reusable capability, high reaction efficiency, low time and superior low temperature.

**Table 3:** Comparison of the performance of the current catalyst with several other catalysts in synthesis 2-amino-4,6-diphenylnicotinonitrile derivatives.

Entry	Reaction conditions	Time (min)	Yield (%)	Lit
1	Yb(PFO)_3_ (2.5 mol%), EtOH, reflux	240	90	^35^
2	Nano Fe_3_O_4_ (30 mg), solvent‐free, 80 °C	120	90	^26^
3	CoFe_2_O_4_@TRIS@ sulfated boric acid (5 mg), Solvent-free, 90 °C	35	90	^21^
4	Cu@imineZCMNPs, solvent-free, 80 °C	18	92	^17^
5	Bu_4_N^+^Br^−^ (10 mol%), H_2_O, reflux	120	92	^18^
6	GO (10), H_2_O, 80 °C	350	96	^36^
7	Fe_3_O_4_@TiO_2_@O_2_PO_2_(CH2)NHSO_3_H, solvent‐free, 90 °C	20	90	^29^
8	Mn@PMO-IL nanocatalyst, solvent free, 80 °C	15–100	55–95	^37^
9	Cellulose–SO_3_H (0.05 mmol), water, 60 °C	150	96	^38^
10	LDH@TRMS@NH_2_SO_2_(C_2_H_4_)SO_2_NH_2_@nano copper, solvent‐free, 60 °C	8	92	This work

## Conclusion

In summary, we present a simple protocol for preparing 2-amino-3-cyanopyridine derivatives using the new green nanocatalyst LDH@TRMS@BDSA@Cu under mild conditions. This synthetic protocol has shown excellent efficiency in conditions such as the absence of solvents, high reaction rate, simple purification, cheap, and cost-effective. The formation of the new nanocatalyst was confirmed by several methods including FT-IR spectroscopy, EDX spectroscopy, elemental mapping, XRD analysis, FESEM, and TGA/DSC and DTA.

## Supplementary Information

Supplementary Information.

## Data Availability

All data generated or analyzed during this study are included in the supplementary information file.

## References

[1] Sun Z (2022). Construction and stabilization of highly active Cu+ sites in layered double hydroxides for the cascade radical addition/cyclization reactions. Mol. Catal..

[2] Ye C (2021). A Mo5N6 electrocatalyst for efficient Na2S electrodeposition in room-temperature sodium-sulfur batteries. Nat. Commun..

[3] Ye C (2020). Electron-state confinement of polysulfides for highly stable sodium—Sulfur batteries. Adv. Mater. Lett..

[4] Roozifar M (2021). Application of salicylic acid as an eco-friendly and efficient catalyst for the synthesis of 2, 4, 6-triaryl pyridine, 2-amino- 3-cyanopyridine, and polyhydroquinoline derivatives. J. Heterocycl. Chem..

[5] Nayak S (2022). Superlative photoelectrochemical properties of 3D MgCr—LDH nanoparticles influencing towards photoinduced water splitting reactions. Sci. Rep..

[6] Ghanbari N, Ghafuri H (2022). Design and preparation of nanoarchitectonics of LDH/polymer composite with particular morphology as catalyst for green synthesis of imidazole derivatives. Sci. Rep..

[7] Kameda T (2021). Kinetic and equilibrium analyses of lactate adsorption by Cu-Al and Mg-Al layered double hydroxides (Cu-Al LDH and Mg-Al LDH) and Cu-Al and Mg-Al layered double oxides (Cu-Al LDO and Mg-Al LDO). Nano-Struct. Nano-Objects.

[8] She QM, Liu JH, Aymonier C, Zhou CH (2021). In situ fabrication of layered double hydroxide film immobilizing gold nanoparticles in capillary microreactor for efficient catalytic carbonylation of glycerol. Mol. Catal..

[9] Arlan FM, Marjani AP, Javahershenas R, Khalafy J (2021). Recent developments in the synthesis of polysubstituted pyridines via multicomponent reactions using nanocatalysts. New J. Chem..

[10] Ding Y, Du X, Zhang X (2022). Rose-like Cu-doped Ni3S2 nanoflowers decorated with thin NiFe LDH nanosheets for high-efficiency overall water and urea electrolysis. Appl. Surf. Sci..

[11] Fu H (2022). Origami and layered-shaped ZnNiFe-LDH synthesized on Cu(OH)2 nanorods array to enhance the energy storage capability. J. Colloid Interface Sci..

[12] Ye C, Chao D, Shan J, Li H, Davey K (2020). Unveiling the advances of 2D materials for Li/Na-S batteries experimentally and theoretically. Matter.

[13] Daraie M, Bagheri D, Malmir M, Heravi MM (2021). Investigation of halloysite nanotubes and Schiff base combination with deposited copper iodide nanoparticles as a novel heterogeneous catalytic system. Sci. Rep..

[14] Chen S, Zhang Y, Zhong H, Cao Z (2022). Trimetallic CoFeCr-LDH@MoS2 as a highly efficient bifunctional electrocatalyst for overall water splitting. Colloids Surf. A Physicochem. Eng. Asp..

[15] Yang C, Kneiß M, Schein F, Lorenz M, Grundmann M (2016). Room-temperature domain- epitaxy of copper iodide thin films for transparent CuI/ZnO heterojunctions with high rectification ratios larger than 10 9. Nat. Publ. Gr..

[16] Yao K (2018). Synthesis of ultrathin two-dimensional nanosheets and van der Waals heterostructures from non-layered γ-CuI. NPJ 2D Mater. Appl..

[17] Yahyazadeh A, Abbaspour-Gilandeh E, Aghaei-Hashjin M (2018). Four-component synthesis of 2-amino-3-cyanopyridine derivatives catalyzed by Cu@imineZCMNPs as a novel, efficient and simple nanocatalyst under solvent-free conditions. Catal. Lett..

[18] Kurumurthy C, Naresh Kumar R, Yakaiah T, Shanthan Rao P, Narsaiah B (2015). Novel Bu4N+Br- catalyzed one-pot multi-component synthesis of 2-amino nicotinonitriles in aqueous medium. Res. Chem. Intermed..

[19] Ismail MMF, Farrag AM, Harras MF, Ibrahim MH, Mehany ABM (2020). Apoptosis: A target for anticancer therapy with novel cyanopyridines. Bioorg. Chem..

[20] Akbarpoor T, Khazaei A, Seyf JY, Sarmasti N, Gilan MM (2019). One-pot synthesis of 2-amino-3-cyanopyridines and hexahydroquinolines using eggshell-based nano-magnetic solid acid catalyst via anomeric-based oxidation. Res. Chem. Intermed..

[21] Faroughi Niya H, Hazeri N, Maghsoodlou MT, Fatahpour M (2021). Synthesis, characterization, and application of CoFe2O4@TRIS@sulfated boric acid nanocatalyst for the synthesis of 2-amino-3-cyanopyridine derivatives. Res. Chem. Intermed..

[22] Zolfigol MA (2017). A convenient method for preparation of 2-amino-4,6-diphenylnicotinonitrile using HBF4 as an efficient catalyst via an anomeric based oxidation: A joint experimental and theoretical study. J. Mol. Struct..

[23] Mojtahedi MM, Hosseinkhany S, Abaee MS, Mesbah AW (2020). A divergent procedure for multicomponent synthesis of novel ferrocenyl derivatives of dicyanoanilines and cyanopyridines. Appl. Organomet. Chem..

[24] Sekhar C (2021). Amberlyst-15 catalysed sonochemical synthesis of 2-amino-4, 6-disubstituted nicotinonitrile derivatives and their biological evaluation. J. Mol. Struct..

[25] Hosseinzadeh Z, Ramazani A, Ahankar H, Ślepokura K, Lis T (2019). Synthesis of 2-amino-4,6-diarylnicotinonitrile in the presence of CoFe2O4@SiO2-SO3H as a reusable solid acid nanocatalyst under microwave irradiation in solvent-freeconditions. SILICON.

[26] Heravi MM, Shirazi SY, Mahzad B (2015). Using magnetic nanoparticles Fe 3 O 4 as a reusable catalyst for the synthesis of pyran and pyridine derivatives via one - pot multicomponent reaction. J. Iran. Chem. Soc..

[27] Mohammadi Ziarani G, Kheilkordi Z, Mohajer F, Badiei A, Luque R (2021). Magnetically recoverable catalysts for the preparation of pyridine derivatives: an overview. RSC Adv..

[28] Motahari, S. & Khazaei, A. Preparation and characterization of Co (II) supported on modified magnetic Fe_3_O_4_ nanoparticles and its application as catalyst for the synthesis of 2-amino-3-cyanopyridines. *Polycycl. Aromat. Compd.* 1–12 (2021).

[29] Zolfigol MA, Yarie M (2017). Fe3O4@TiO2@O2PO2(CH2)NHSO3H as a novel nanomagnetic catalyst: Application to the preparation of 2-amino-4,6-diphenylnicotinonitriles via anomeric-based oxidation. Appl. Organomet. Chem..

[30] Asadbegi S, Bodaghifard MA, Mobinikhaledi A (2020). Poly N, N-dimethylaniline-formaldehyde supported on silica-coated magnetic nanoparticles: a novel and retrievable catalyst for green synthesis of 2-amino-3-cyanopyridines. Res. Chem. Intermed..

[31] Khalifeh R, Ghamari M (2016). A multicomponent synthesis of 2-amino-3-cyanopyridine derivatives catalyzed by heterogeneous and recyclable copper nanoparticles on charcoal. J. Braz. Chem. Soc..

[32] Afradi M, Pour SA, Dolat M, Yazdani-Elah-Abadi A (2018). Nanomagnetically modified vitamin B3 (Fe3O4@Niacin): An efficient and reusable green biocatalyst for microwave-assisted rapid synthesis of 2-amino-3-cyanopyridines in aqueous medium. Appl. Organomet. Chem..

[33] Ghorbani-Vaghei R, Jalili H (2005). Mild. regioselective bromination of aromatic compounds with N, N, N′, N′-tetrabromobenzene-1,3-disulfonylamide and poly(N-bromobenzene-1,3-disulfonylamide). Synthesis (Stuttg).

[34] Rohani S, Ziarati A (2012). CuI nanoparticles as a reusable heterogeneous catalyst for the one-pot synthesis of N-cyclohexyl-3-aryl-quinoxaline-2- amines under mild conditions. J. Nanostruct..

[35] Wang L (2005). Facile Yb (OTf) 3 promoted one-pot synthesis of polyhydroquinoline derivatives through Hantzsch reaction. Tetrahedron Lett..

[36] Kalili D (2016). Graphene oxide: a reusable and metal-free carbocatalyst for the one-pot synthesis of 2-amino-3-cyanopyridines in water. Tetrahedron Lett..

[37] Nasr-Esfahani M, Elhamifar D, Amadeh T, Karimi B (2015). Periodic mesoporous organosilica with ionic-liquid framework supported manganese: an efficient and recyclable nanocatalyst for the unsymmetric Hantzsch reaction. RSC Adv..

[38] Mansoor SS, Aswin K, Logaiya K, Sudhan PN, Malik S (2014). Aqueous media preparation of 2-amino-4,6-diphenylnicotinonitriles using cellulose sulfuric acid as an efficient catalyst. Res. Chem. Intermed..

