# Supplementary material for: Synthesis, properties, and application of the new nanocatalyst of double layer hydroxides in the one-pot multicomponent synthesis of 2-amino-3-cyanopyridine derivatives
Source: Sci Rep. 2023 Jan 28;13:1627. doi: 10.1038/s41598-023-27940-6 (PMC9884200; doi:10.1038/s41598-023-27940-6)
Supplement: Supplementary file 1 — Supplementary Information. [file 41598_2023_27940_MOESM1_ESM.docx]

**Supporting Information**

**(Scientific Reports)**

**Synthesis, properties, and application of the new nanocatalyst of double layer hydroxides in the one-pot multicomponent synthesis of 2-amino-3-cyanopyridine derivativesp**

Sarieh Momeni, Ramin Ghorbani-Vaghei*

*Department of Organic Chemistry, Faculty of Chemistry, Bu-Ali Sina University, Hamedan, 6517838683, Iran*

**Corresponding author; E-mail:* [*rgvaghei@yahoo.com*](mailto:rgvaghei@yahoo.com) *&* [*ghorbani@basu.ac.ir*](mailto:ghorbani@basu.ac.ir)

Contents Pages

[**Spectra data** ..3](#_Toc97457812)

[2-amino-4-(4-chlorophenyl)-6-cyclopropylnicotinonitrile (5g) 3](#_Toc97457814)

2-amino-6-cyclopropyl-4-(2,4-dichlorophenyl)nicotinonitrile (5k)……………………………3

[2-amino-6-cyclopropyl-4-(4-methoxyphenyl)nicotinonitrile (5h) 3](#_Toc97457815)

[2-amino-6-cyclopropyl-4-(2-fluorophenyl)nicotinonitrile (5i) 3](#_Toc97457816)

[2-amino-4-(4-fluorophenyl)-6-phenylnicotinonitrile (5d) 3](#_Toc97457817)

Figure 1. [HNMR Spectrum of 2-amino-4-(4-chlorophenyl)-6-](#_Toc97457829)

[cyclopropylnicotinonitrile (5g). 4](#_Toc97457829)

Figure 2. [CNMR Spectrum of 2-amino-4-(4-chlorophenyl)-6-](#_Toc97457830)

[cyclopropylnicotinonitrile (5g)](#_Toc97457830)*[.](#_Toc97457830)* [5](#_Toc97457830)

Figure 3. [FTIR Spectrum of 2-amino-4-(4-chlorophenyl)-6-](#_Toc97457831)

[cyclopropylnicotinonitrile (5g). 6](#_Toc97457831)

Figure 4. [HNMR Spectrum of 2-amino-6-cyclopropyl-4-](#_Toc97457832)

[(2,4-dichlorophenyl)nicotinonitrile (5k). 7](#_Toc97457832)

Figure 5. [CNMR Spectrum of 2-amino-6-cyclopropyl-4-](#_Toc97457833)

[(2,4-dichlorophenyl)nicotinonitrile (5k). 8](#_Toc97457833)

Figure 6. [FTIR Spectrum of 2-amino-6-cyclopropyl-4-](#_Toc97457834)

[(2,4-dichlorophenylFT)nicotinonitrile (5k). ……………..…….9](#_Toc97457834)

Figure 7. [HNMR Spectrum of 2-amino-6-cyclopropyl-4-](#_Toc97457835)

[(4-methoxyphenyl)nicotinonitrile (5h) 10](#_Toc97457835)

Figure 8. [CNMR Spectrum of 2-amino-6-cyclopropyl-4-](#_Toc97457836)

[(4-methoxyphenyl)nicotinonitrile (5h) 11](#_Toc97457836)

Figure 9. [FTIR Spectrum of](#_Toc97457837)[2-amino-6-cyclopropyl-4-](#_Toc97457837)

[(4-methoxyphenyl)nicotinonitrile (5h). 12](#_Toc97457837)

Figure 10. [HNMR Spectrum of 2-amino-6-cyclopropyl-4-](#_Toc97457838)

[(2-fluorophenyl)nicotinonitrile in chloroform solvent (5i)……………………….……………...…13](#_Toc97457838)

Figure 11. [HNMR Spectrum of 2-amino-6-cyclopropyl-4-](#_Toc97457839)

[(2-fluorophenyl)nicotinonitrile in DMSO solvent(5i)…………………….………….….14](#_Toc97457839)

Figure 12. [CNMR Spectrum of 2-amino-6-cyclopropyl-4-](#_Toc97457840)

[(2-fluorophenyl)nicotinonitrile in DMSO solvent (5i).…….................................................15](#_Toc97457840)

Figure 13. FTIR Spectrum of 2-amino-6-cyclopropyl-4-

(2-fluorophenyl)nicotinonitrile(5i)……………………… ………………………………16

Figure 14. [HNMR Spectrum of 2-amino-4-(4-fluorophenyl)-6-](#_Toc97457841)

[phenylnicotinonitrile(5d)…](#_Toc97457841)…………………………..………………………………….17

Figure 15. [CNMR Spectrum of 2-amino-4-(4-fluorophenyl)-6-](#_Toc97457842)

[phenylnicotinonitrile (5d)](#_Toc97457842) ………………………………………………………………18

Figure 16. [FTIR Spectrum of 2-amino-4-(4-fluorophenyl)-6-](#_Toc97457843)

[phenylnicotinonitrile(5d)…..](#_Toc97457843)...................……………………………………………….19

**Spectra data**

*2-amino-4-(4-chlorophenyl)-6-cyclopropylnicotinonitrile (5g)*

M.p. 178-180 °C, FT-IR (KBr, ν cm^-1^): 3419, 3317, 3168, 2208, 1646, 1495, 1382, 1257, 818. 1H NMR (300 MHz, Chloroform-d): δ (ppm) 7.60-7.39 (s, 5H), 6.54 (s, 1H), 5.16 (s, 1H), 1.94 (s, 1H), 1.08 (s, 4H). 13C NMR (63 MHz, DMSO): δ (ppm) 167.56, 161.40, 152.75, 136.07, 135.17, 130.47, 129.34, 118.02, 111.36, 85.07, 17.71, 10.75.

*2-amino-6-cyclopropyl-4-(2,4-dichlorophenyl)nicotinonitrile (5k)*

M.p. 156-158 °C, FT-IR (KBr, ν cm^-1^): 3407, 3314, 3175, 2211, 1647, 1557, 1471, 1389, 1267, 1101, 818. 1H NMR (300 MHz, Chloroform-d): δ (ppm) 7.57 (s, 1H), 7.35 (s, 2H), 6.57 (s, 1H), 5.09 (s, 2H), 1.97 (s, 1H), 1.13 – 0.99 (m, 4H). 13C NMR (63 MHz, DMSO): δ (ppm) 167.82, 160.93, 151.27, 135.64, 134.94, 132.86, 129.61, 128.14, 116.93, 111.54, 86.73, 17.73, 10.95.

*2-amino-6-cyclopropyl-4-(4-methoxyphenyl)nicotinonitrile (5h)*

M.p. 128-130 °C, FT-IR (KBr, ν cm^-1^): 3433, 3351, 3238, 2211, 1640, 1574, 1455, 1382, 1249, 828. 1H NMR (250 MHz, DMSO-d6): δ (ppm) 7.52 (d, 2H), 7.02 (d, 2H), 7.00 – 6.60 (m, 2H), 6.59 (s, 1H), 3.87 (s, 3H), 1.97 (s, 1H), 1.06 (d, J = 65.4 Hz, 4H). 13C NMR (63 MHz, DMSO): δ (ppm) 167.14, 161.19, 160.50, 153.65, 130.02, 117.80, 114.57, 110.91, 102.92, 85.29, 55.50, 17.66, 11.30.

*2-amino-6-cyclopropyl-4-(2-fluorophenyl)nicotinonitrile (5i)*

M.p. 132-134 °C, FT-IR (KBr, ν cm^-1^): 3409, 3311, 3164, 2215, 1647, 1494, 1389, 1258,1000, 881, 756. 1H NMR (300 MHz, DMSO-d6): δ (ppm) 7.50 – 7.31 (m, 4H, ArH), 6.81 (s, 2H, NH2), 6.63 (s, 1H, pyridine ring), 2.01 (p, J = 6.4 Hz, 1H, C-H), 0.96 (d, J = 6.4 Hz, 4H, 2 C-H2). 13C NMR (63 MHz, DMSO): δ (ppm) 167.56, 160.98, 148.92, 132.40, 131.22, 125.28, 116.62, 116.14, 111.57, 86.73, 17.68, 10.82.

*2-amino-4-(4-fluorophenyl)-6-phenylnicotinonitrile (5d)*

M.p. 208-211 °C, FT-IR (KBr, ν cm^-1^): 3475, 3311, 3195, 2207, 1645, 1575, 1511, 1448, 1372, 1234, 1159, 831, 767. 1H NMR (300 MHz, Chloroform-d): δ (ppm) 8.00 (dt, J = 6.3, 4.0 Hz, H), 7.64 (s, 2H), 7.50 (s, 2H), 7.22 (d, J = 8.6 Hz, 2H), 5.39 (s, 2H), 1.26 (s, 1H), 0.93 (s, 4H).


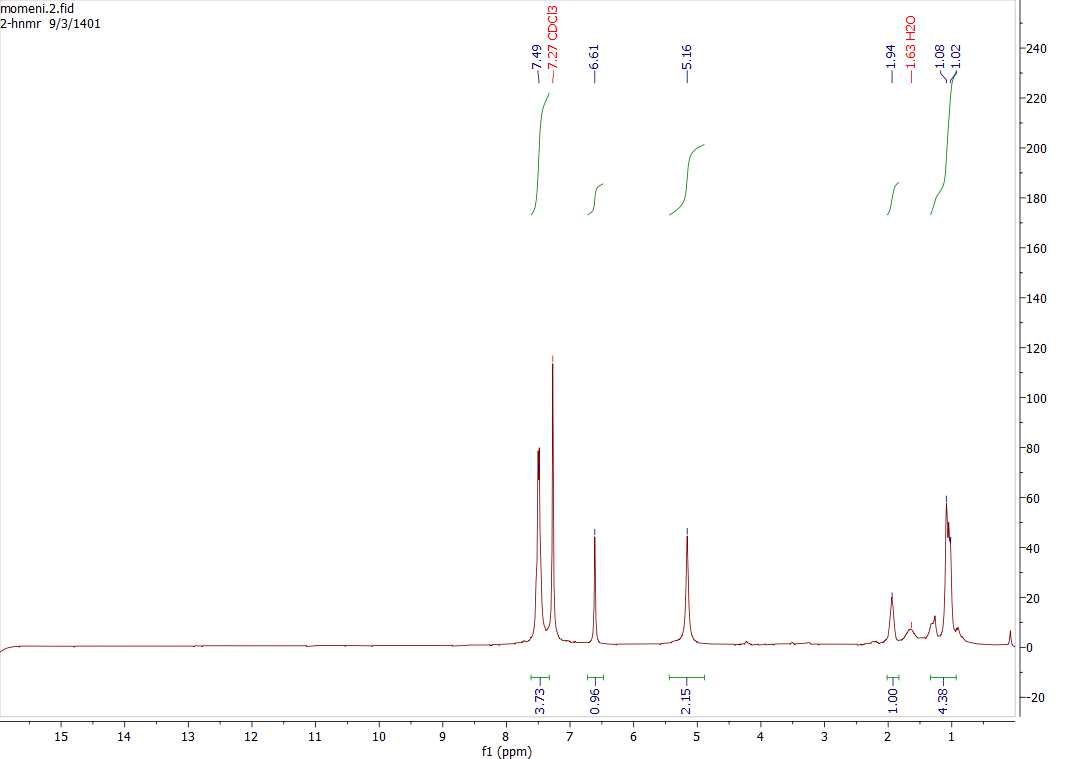


Figure 1. HNMR Spectrum of *2-amino-4-(4-chlorophenyl)-6-cyclopropylnicotinonitrile* (5g)


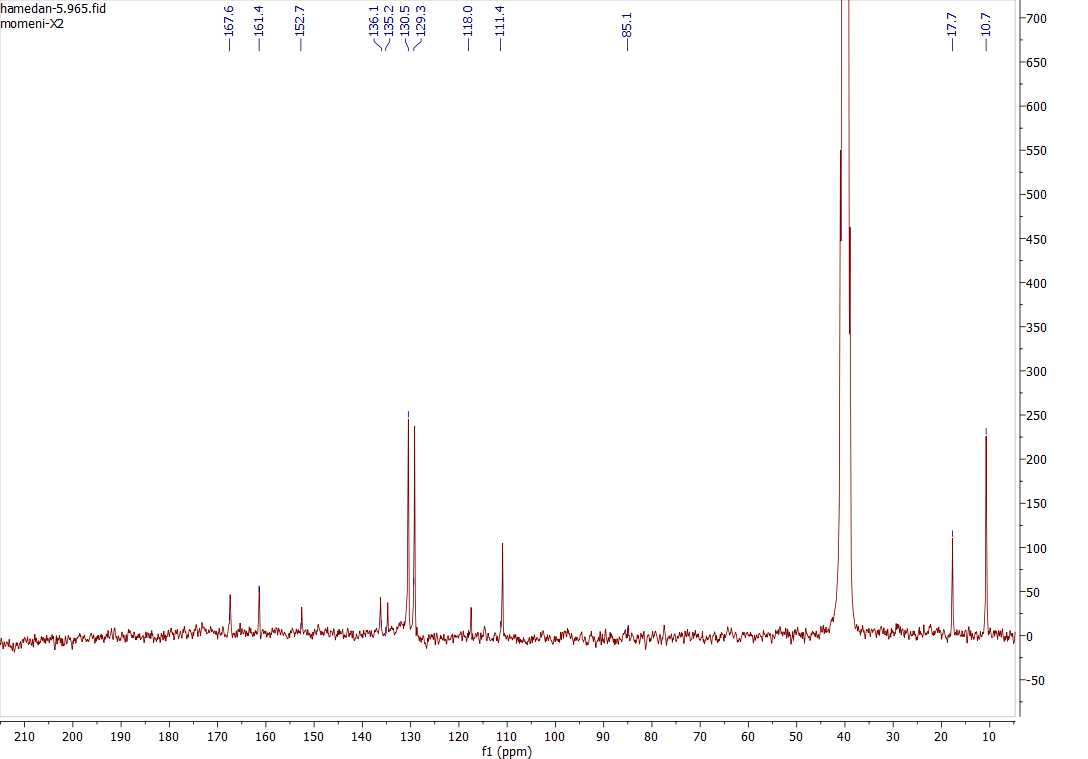


Figure 2. CNMR Spectrum of *2-amino-4-(4-chlorophenyl)-6-cyclopropylnicotinonitrile* (5g)


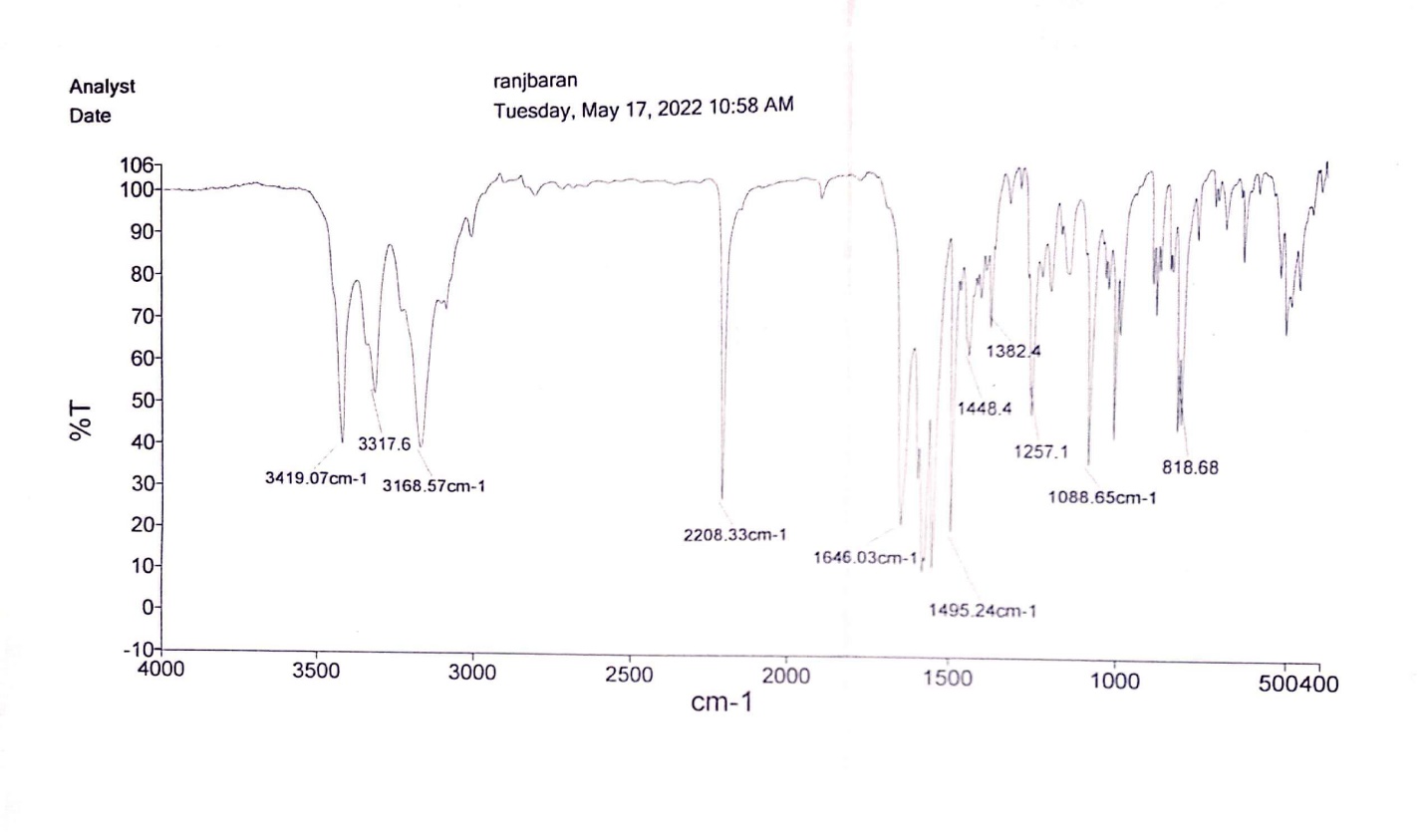


Figure 3. FTIR Spectrum of *2-amino-4-(4-chlorophenyl)-6-cyclopropylnicotinonitrile* (5g)


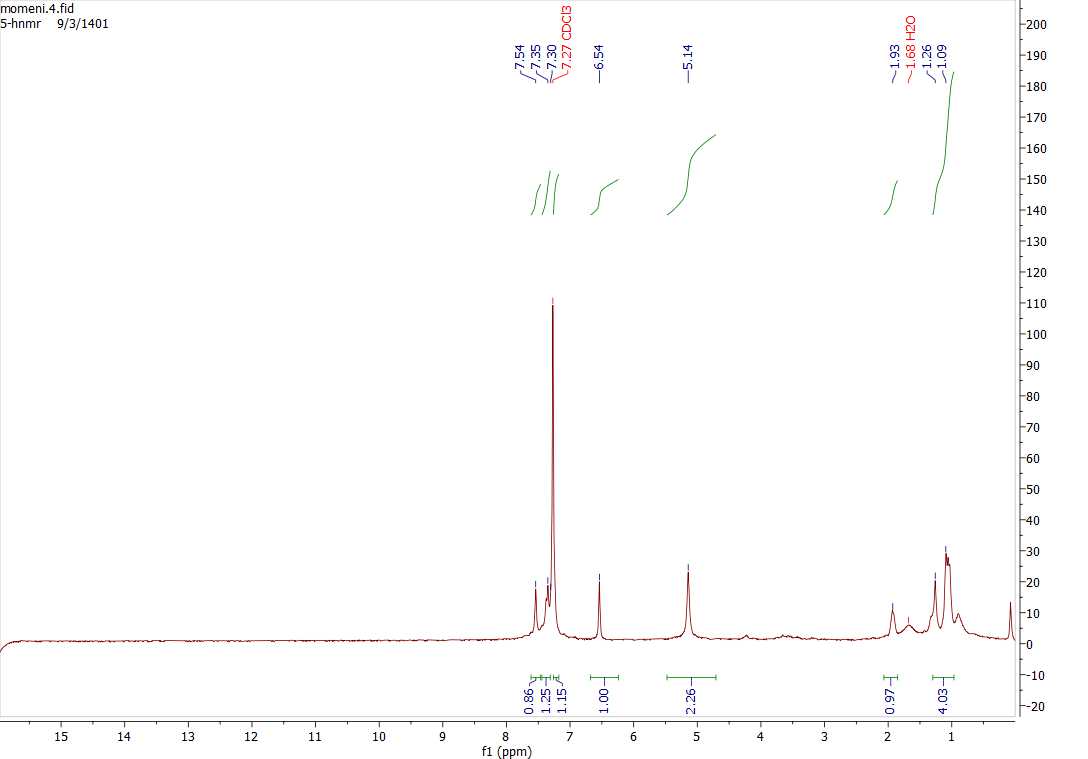


Figure 4. HNMR Spectrum of *2-amino-6-cyclopropyl-4-(2,4-dichlorophenyl)nicotinonitrile* (5k)


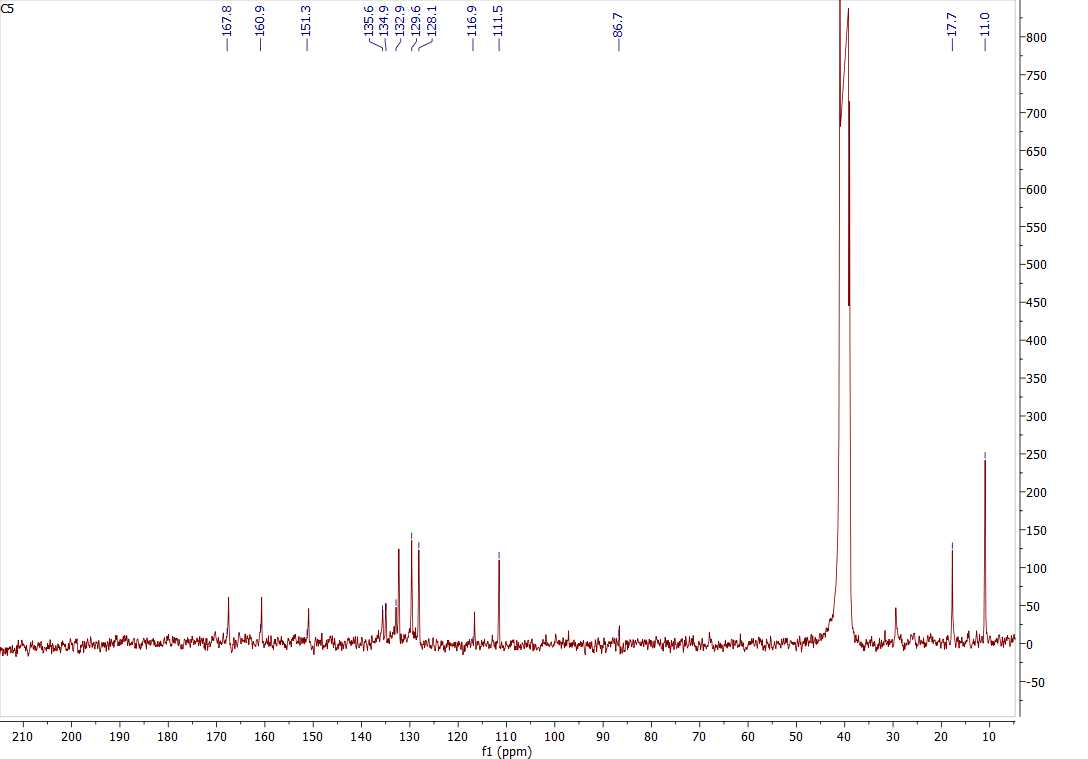

Figure 5. CNMR Spectrum of *2-amino-6-cyclopropyl-4-(2,4-dichlorophenyl)nicotinonitrile* (5k)


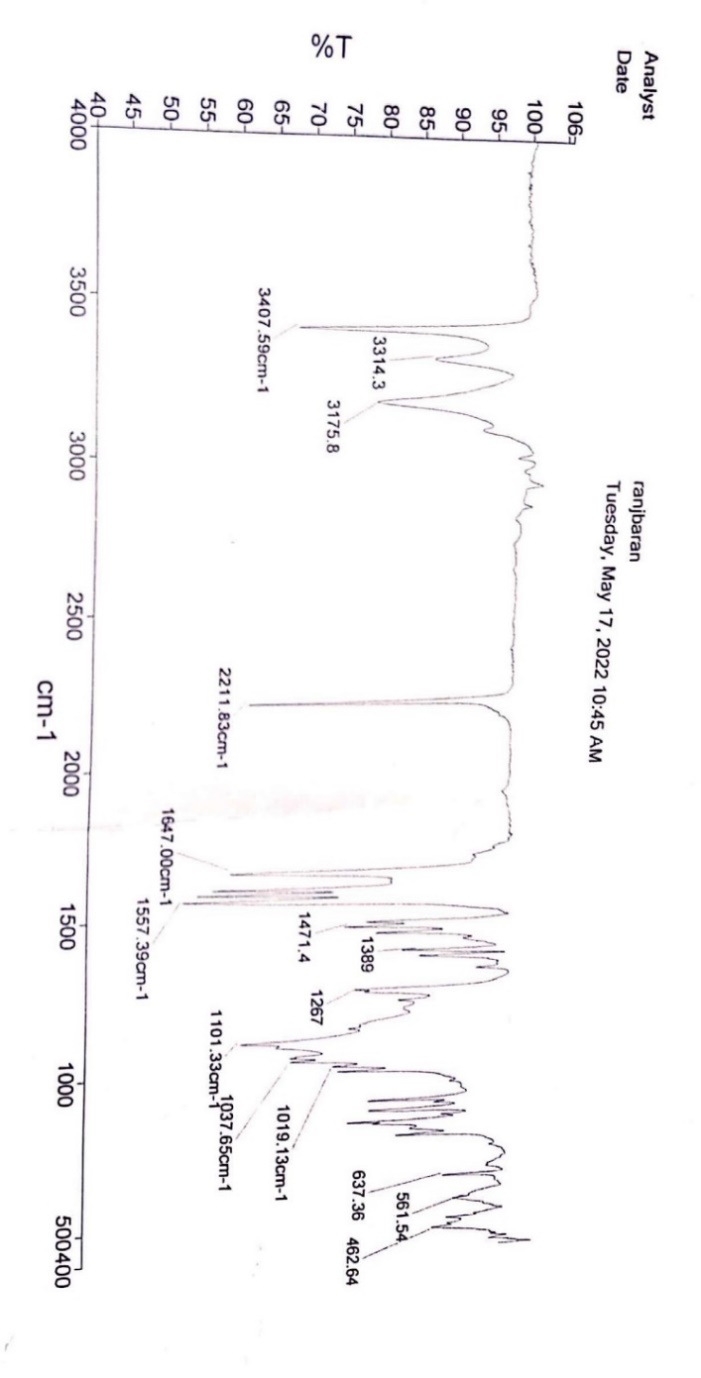

Figure 6. FTIR Spectrum of *2-amino-6-cyclopropyl-4-(2,4-dichlorophenyl)nicotinonitrile* (5k)


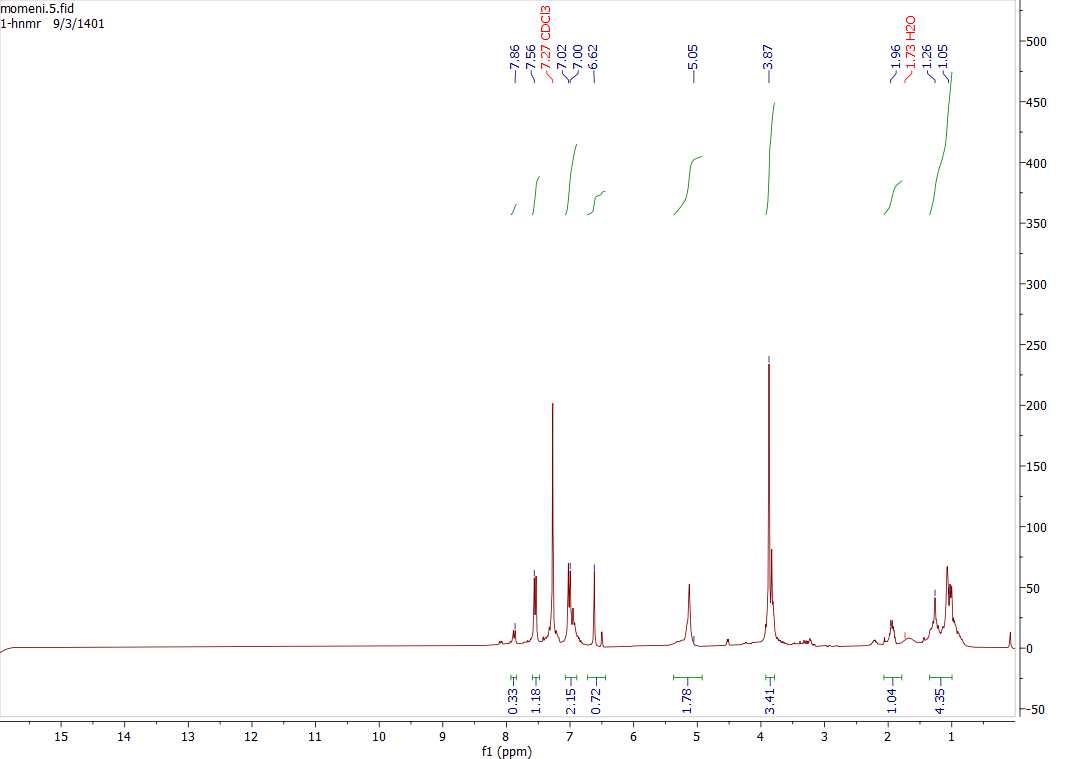


Figure 7. [HNMR Spectrum of *2-amino-6-cyclopropyl-4-(4-methoxyphenyl)nicotinonitrile* (5h)](#_Toc97457833)


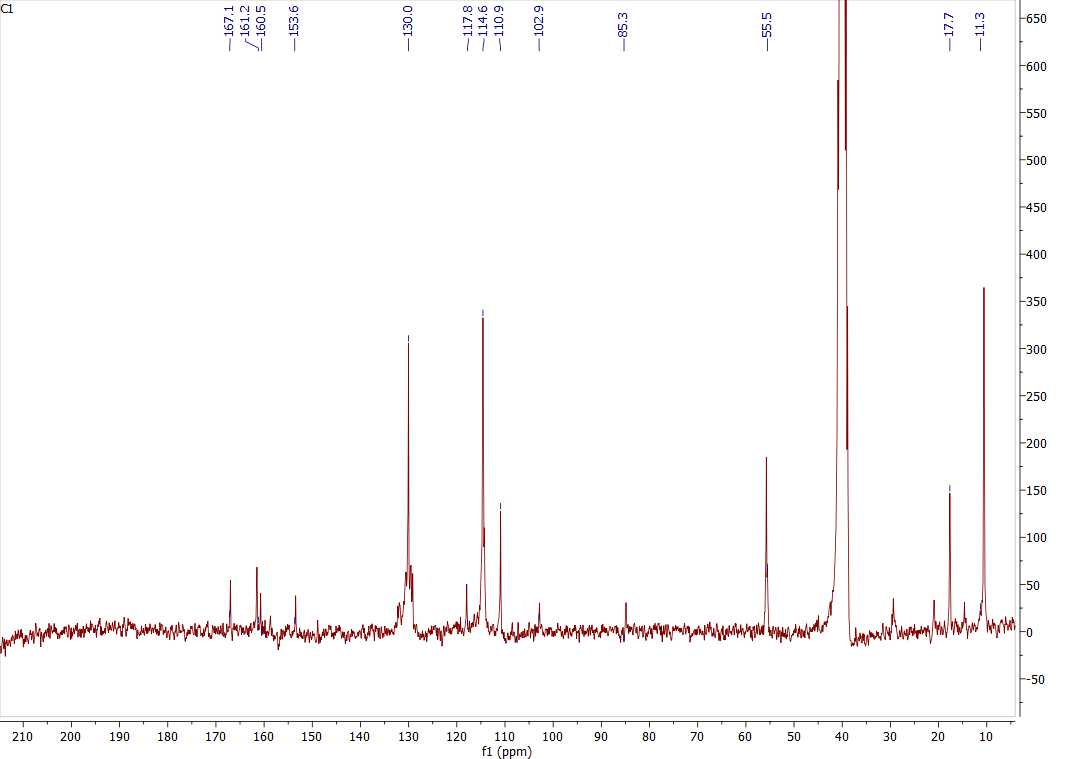

Figure 8. [CNMR Spectrum of *2-amino-6-cyclopropyl-4-(4-methoxyphenyl)nicotinonitrile* (5h)](#_Toc97457833)


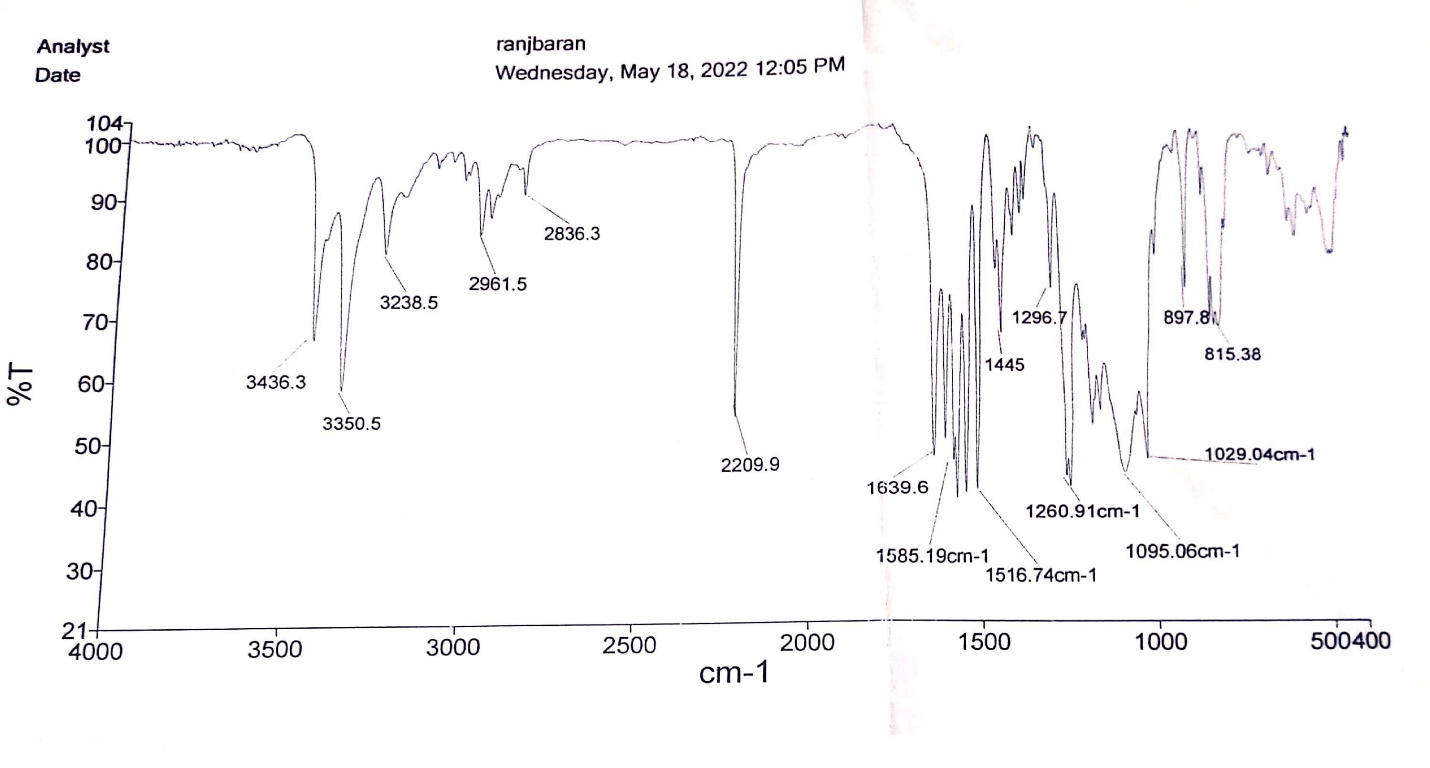


Figure 9. [FTIR Spectrum of *2-amino-6-cyclopropyl-4-(4-methoxyphenyl)nicotinonitrile* (5h)](#_Toc97457833)


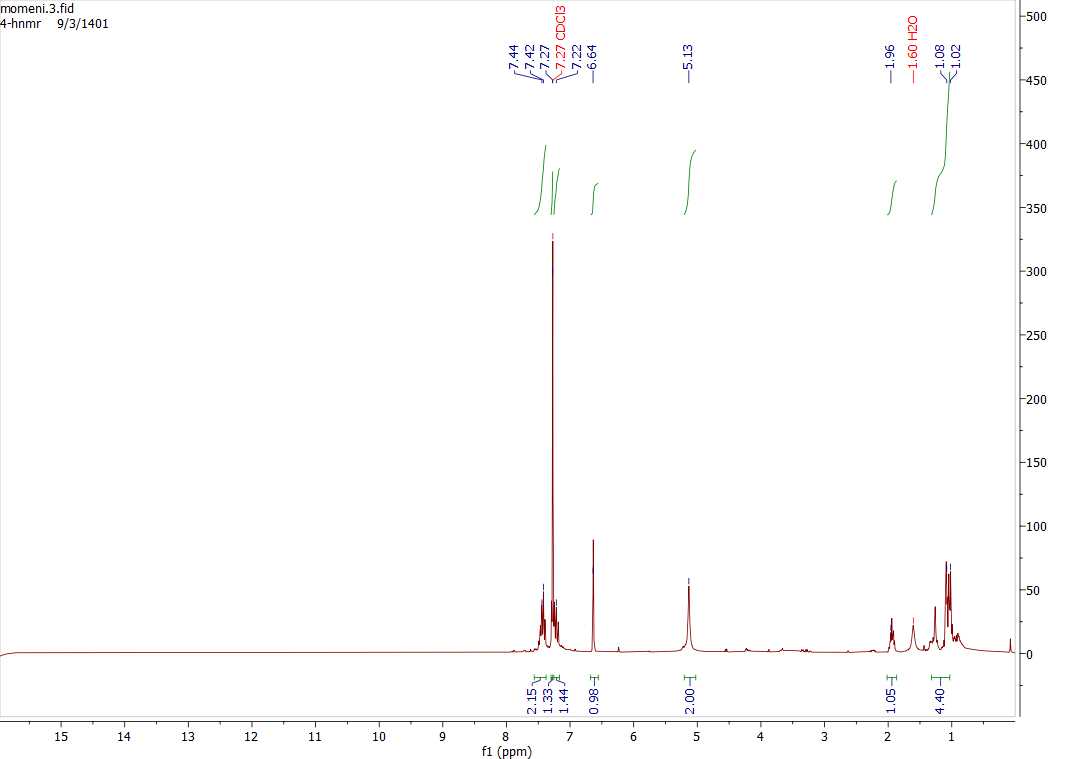


Figure 10. HNMR Spectrum of *2-amino-6-cyclopropyl-4-(2-fluorophenyl)nicotinonitrile* in chloroform solvent (5i)


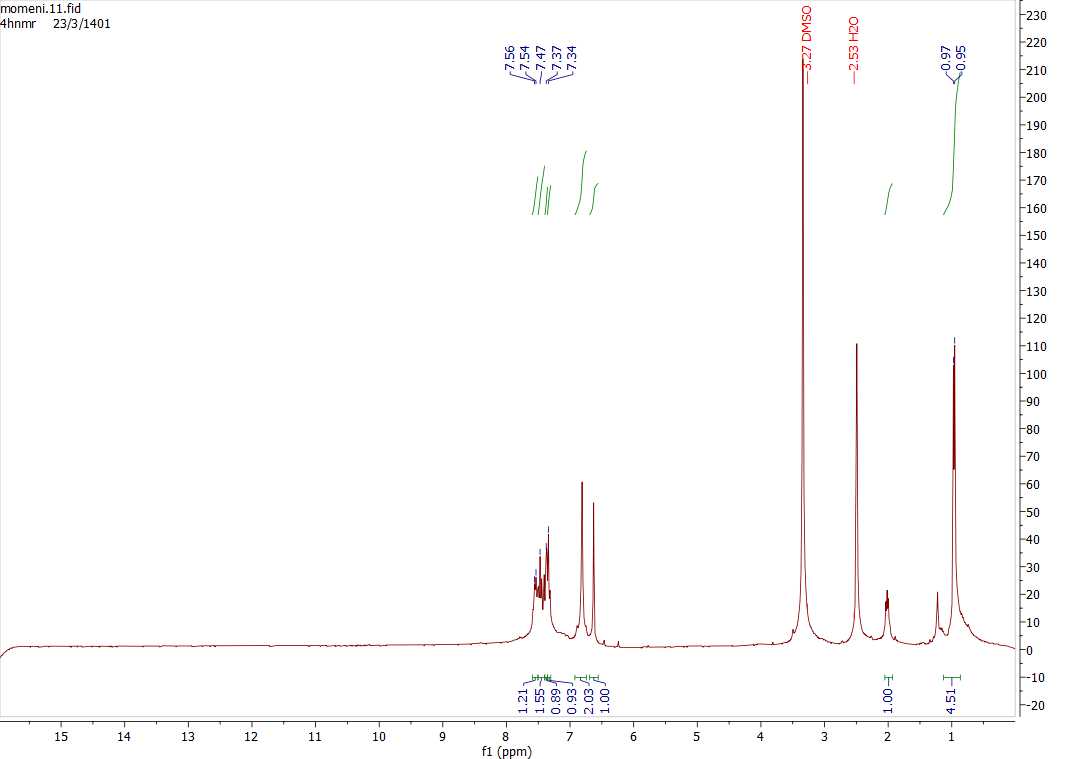

Figure 11. HNMR Spectrum of *2-amino-6-cyclopropyl-4-(2-fluorophenyl)nicotinonitrile* in DMSO solvent (5i)


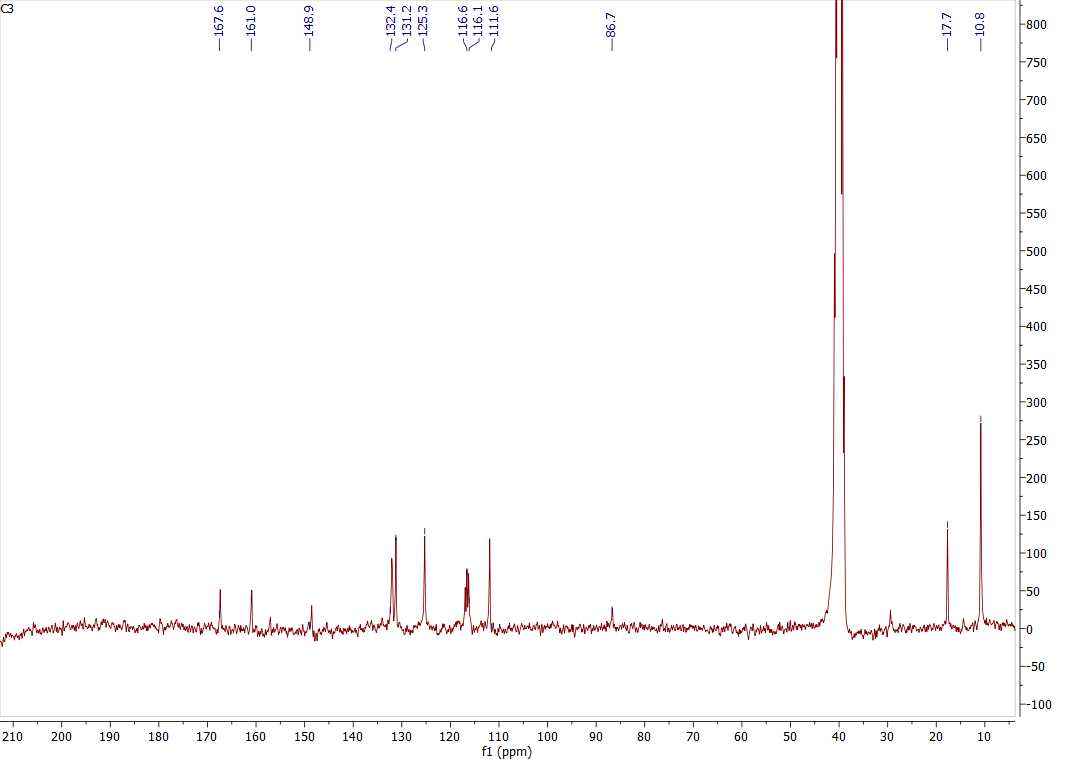


Figure 12. CNMR Spectrum of *2-amino-6-cyclopropyl-4-(2-fluorophenyl)nicotinonitrile* in DMSO solvent (5i)


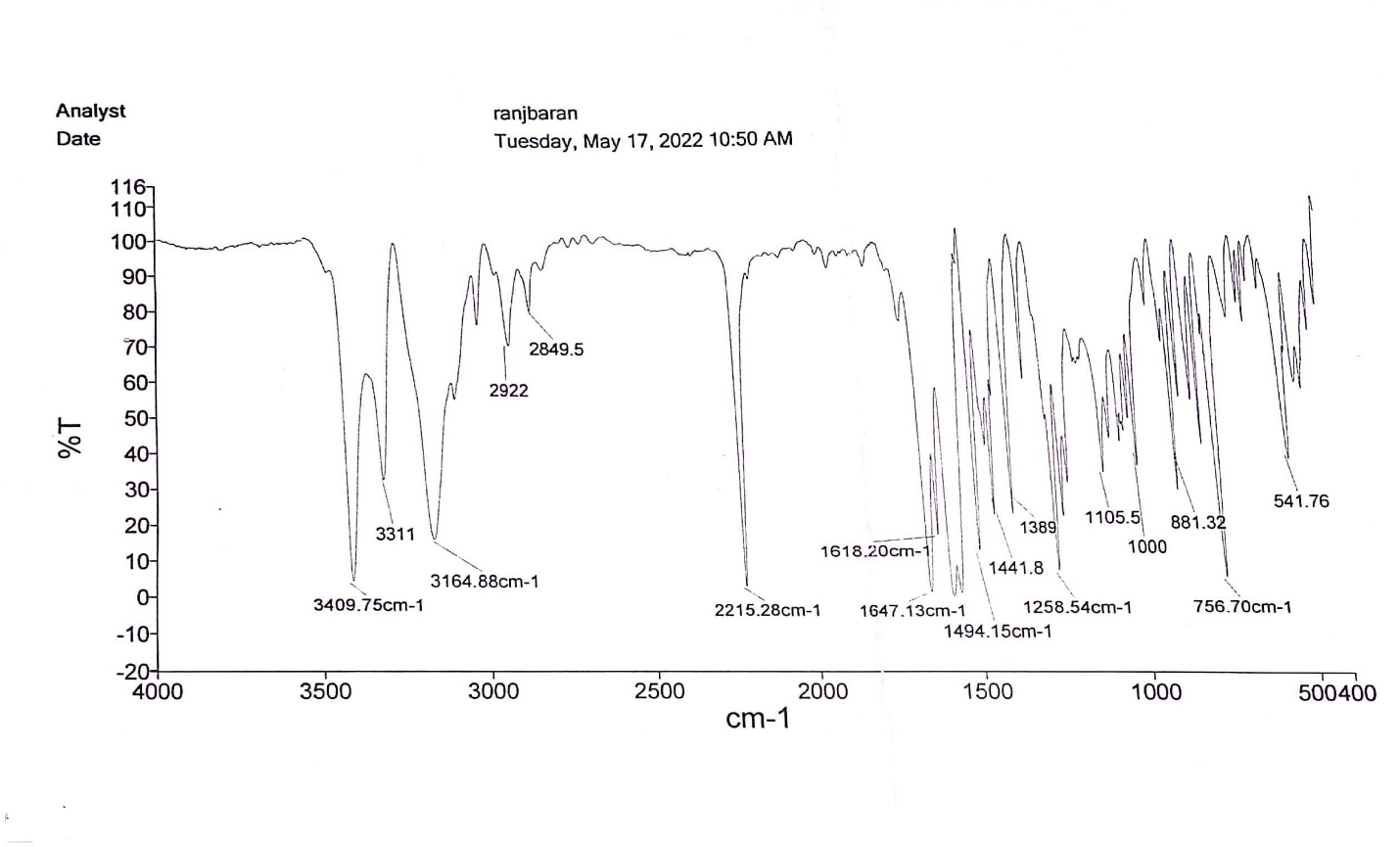

Figure 13. FTIR Spectrum of *2-amino-6-cyclopropyl-4-(2-fluorophenyl)nicotinonitrile* (5i)


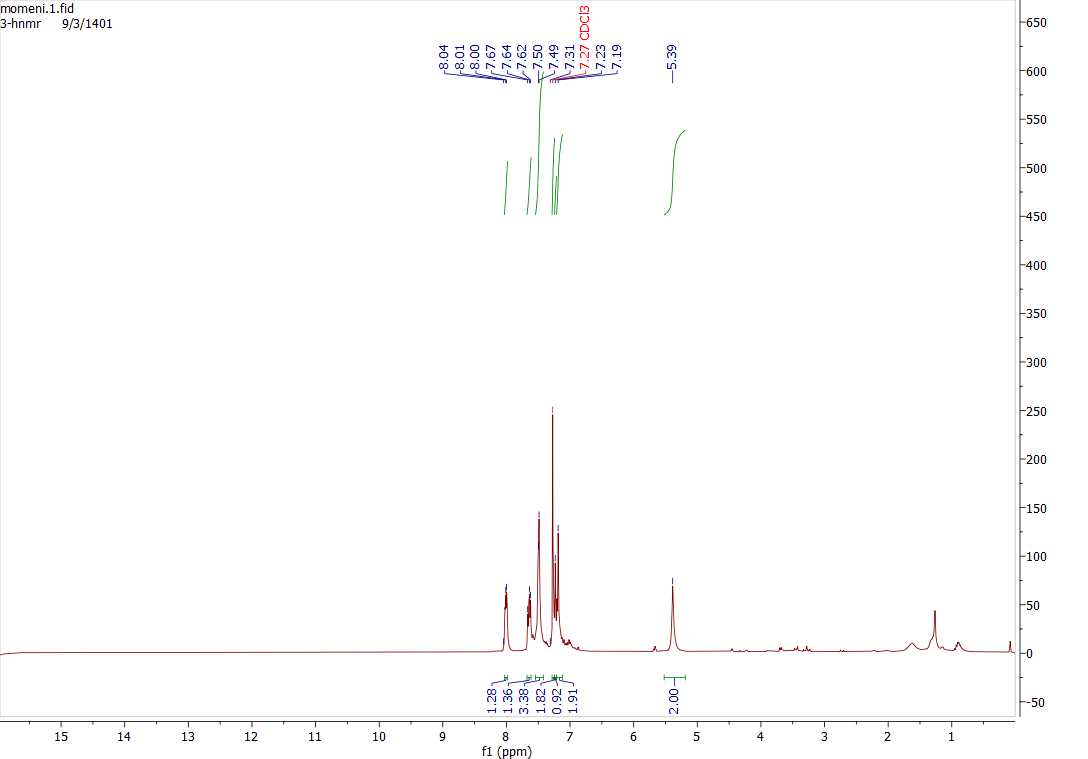


Figure 14. HNMR Spectrum of *2-amino-4-(4-fluorophenyl)-6-phenylnicotinonitrile* (5d)


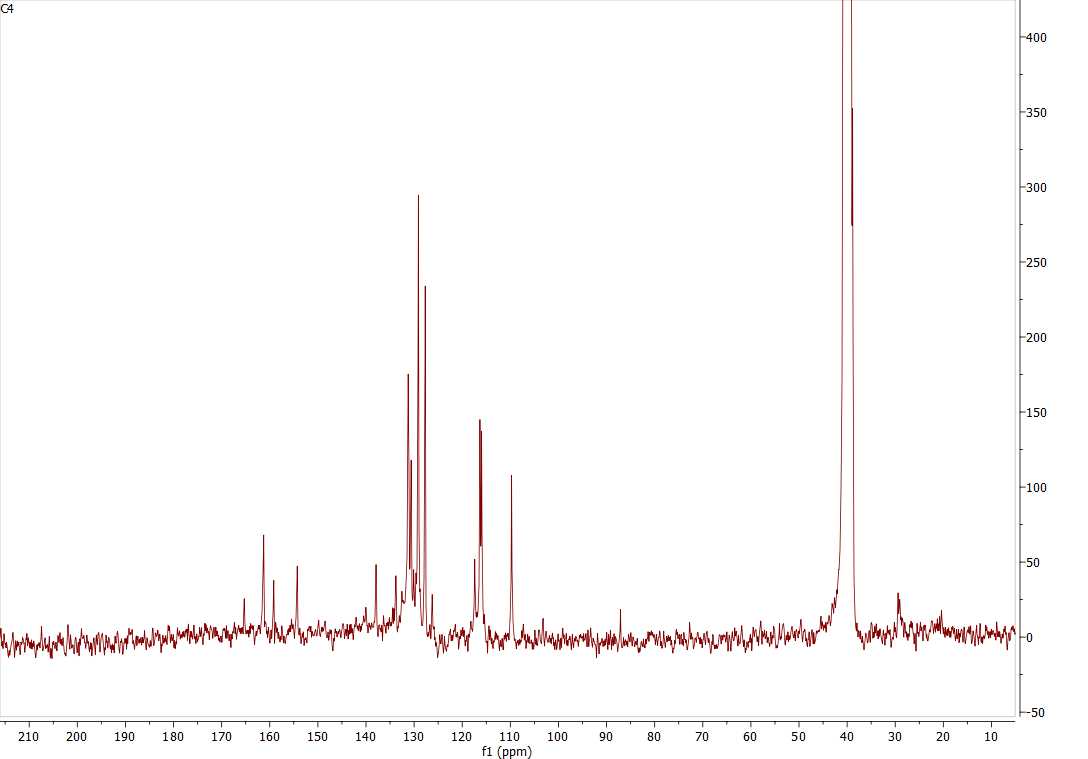


Figure 15. CNMR Spectrum of *2-amino-4-(4-fluorophenyl)-6-phenylnicotinonitrile* (5d)


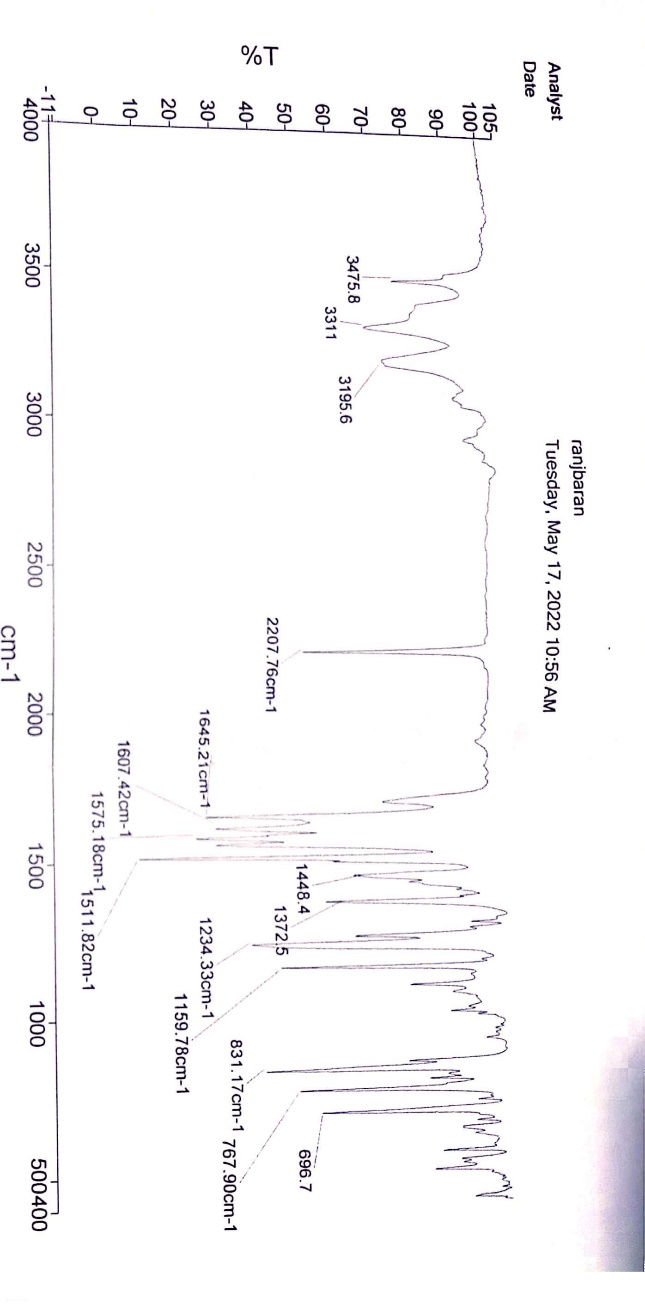


Figure 16. FTIR Spectrum of *2-amino-4-(4-fluorophenyl)-6-phenylnicotinonitrile* (5d)
